# Supplementary material for: Leveraging Public Data to Predict Global Niches and Distributions of Rhizostome Jellyfishes
Source: Animals (Basel). 2023 May 9;13(10):1591. doi: 10.3390/ani13101591 (PMC10215779; doi:10.3390/ani13101591)
Supplement: Supplementary file 1 [file animals-13-01591-s001.zip › Anthonyetal_Animals_File_S1_SupplementalFigures.pdf]

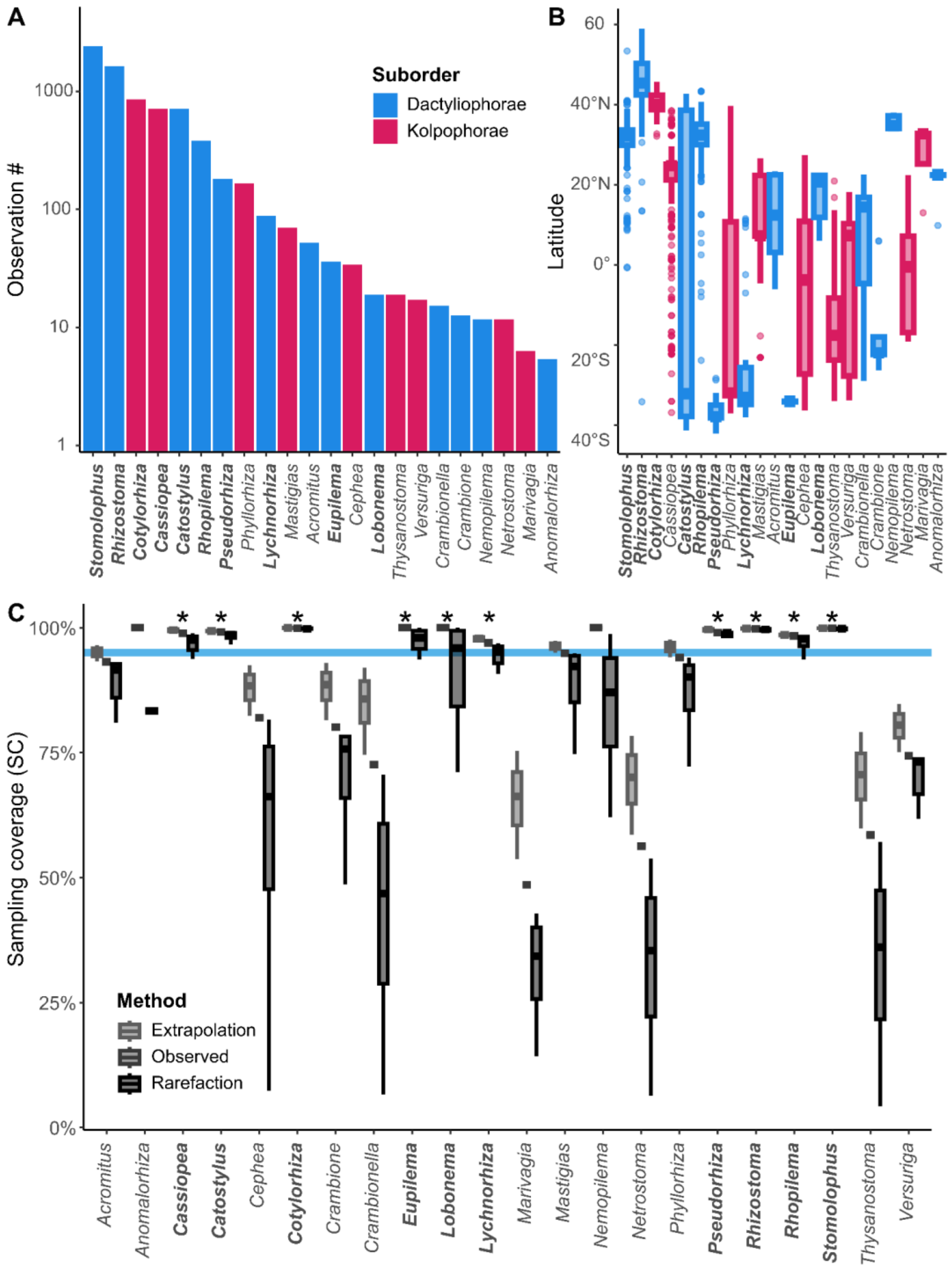

Figure S1. Observation abundance (**A**) and coordinate diversity (**B**) inform the percentage of sampling coverage (**C**). Genera with >95% rarefied sampling coverage (*) were maintained for niche and distribution models.

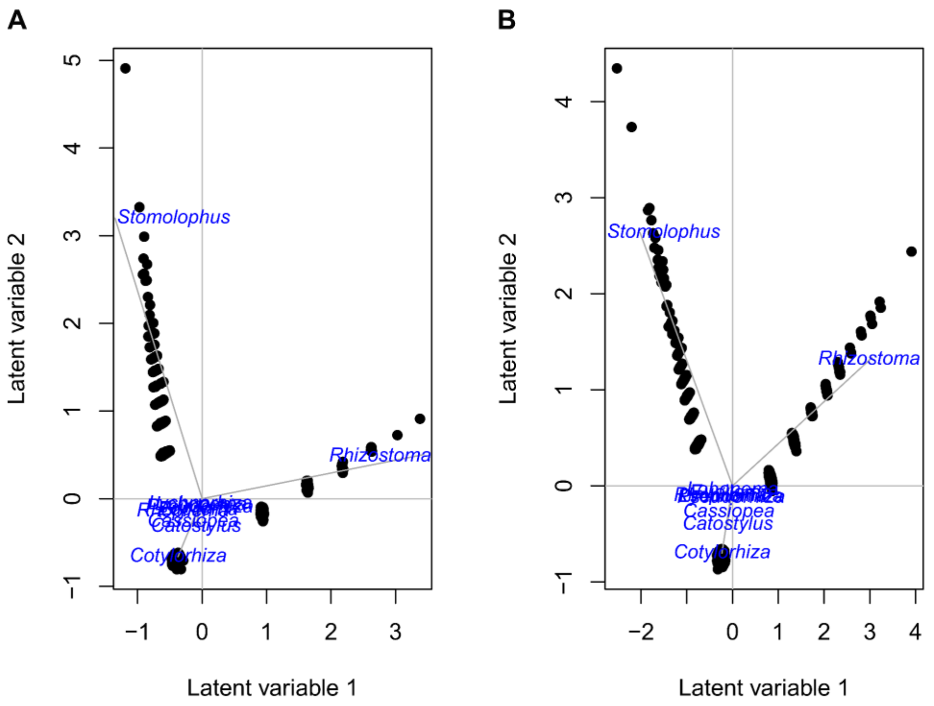

Figure S2. Multivariate GLM niche models did not change when environmental data was mapped to iNaturalist occurrence data by different coordinate scales: 1° (**A**) and 2° (**B**) latitudinal and longitudinal mapping.

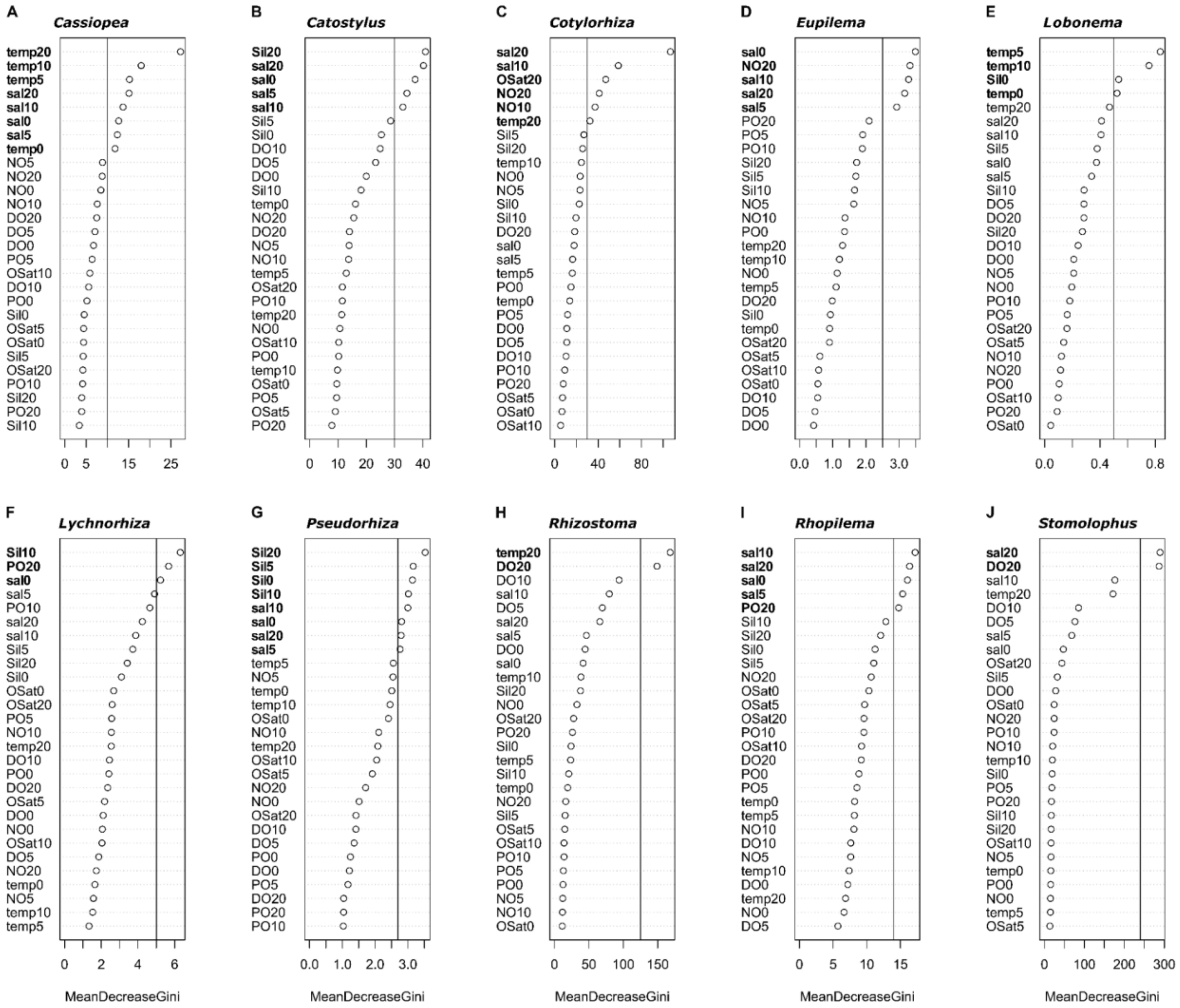

Figure S3. Environmental predictors are ranked by importance to RFMs (Mean Decrease Gini) for each genus (**A-J**). Lines indicate importance thresholds used to predict genus distributions (Figure S4).

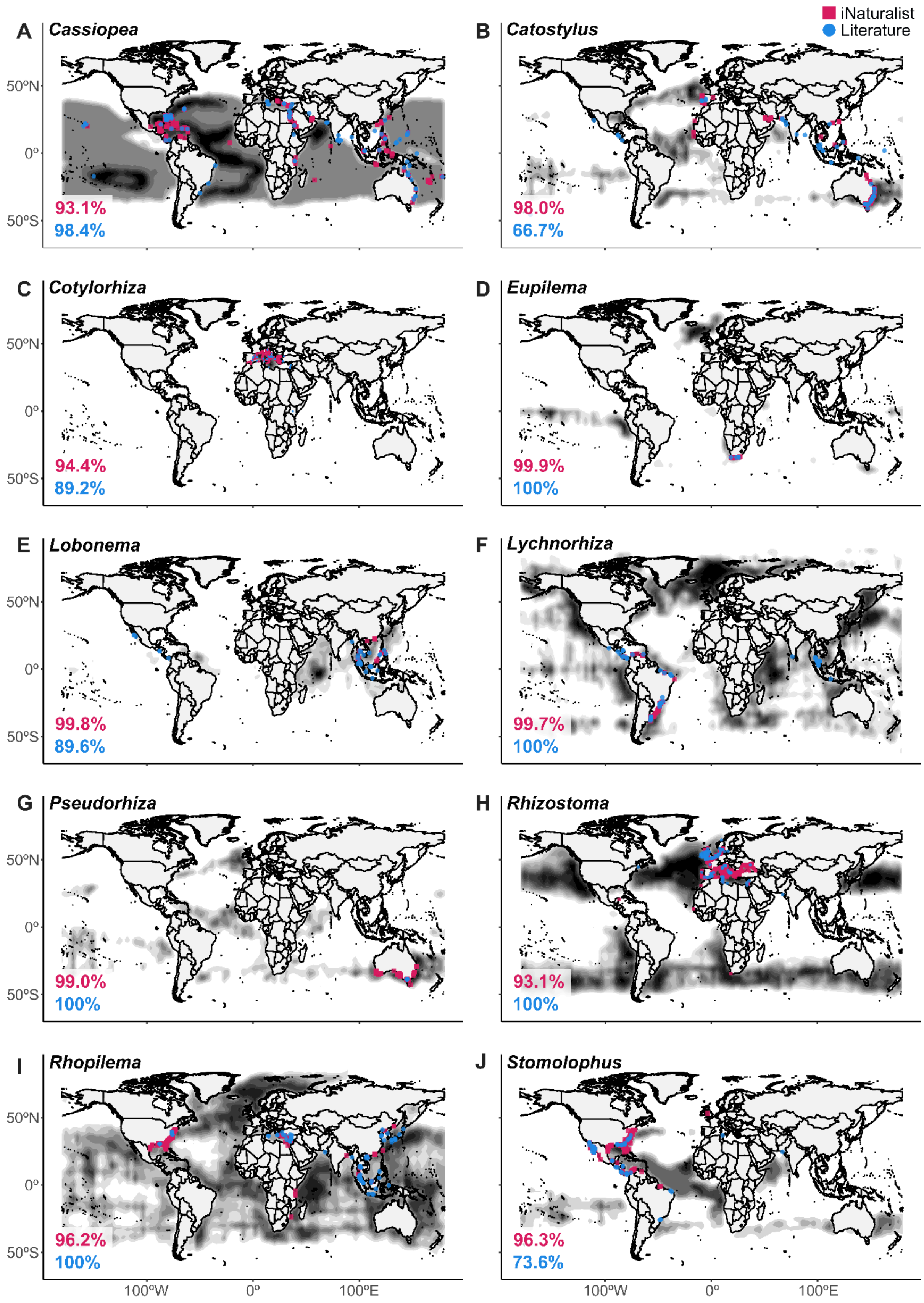
iNaturalist
Literature
A
Cassiopea
93.1%
98.4%
B
Catostylus
98.0%
66.7%
C
Cotylorhiza
94.4%
89.2%
D
Eupilema
99.9%
100%
E
Lobonema
99.8%
89.6%
F
Lychnorhiza
99.7%
100%
G
Pseudorhiza
99.0%
100%
H
Rhizostoma
93.1%
100%
I
Rhopilema
96.2%
100%
J
Stomolophus
96.3%
73.6%
50ºN
0º
50ºS
100ºW
0º
100ºE

Figure S4. Pink squares indicate iNaturalist observations used to build distribution models, while blue circles indicate observations from the literature. Dark density-based polygons indicate predicted environments capable of supporting the genus based on random forest models (Figure 2B; S3) for *Cassiopea* (**A**), *Catostylus* (**B**), *Cotylorhiza* (**C**), *Eupilema* (**D**), *Lobonema* (**E**), *Lychnorhiza* (**F**), *Pseudorhiza* (**G**), *Rhizostoma* (**H**), *Rhopilema* (**I**), *Stomolophus* (**J**). Pink percentages reflect RFM model accuracy built from all iNaturalist observations, while blue percentages estimate the distribution model accuracy based on observations from published literature (File S2).
