# Supplementary material for: Leveraging Public Data to Predict Global Niches and Distributions of Rhizostome Jellyfishes
Source: Animals (Basel). 2023 May 9;13(10):1591. doi: 10.3390/ani13101591 (PMC10215779; doi:10.3390/ani13101591)
Supplement: Supplementary file 1 [file animals-13-01591-s001.zip › Anthonyetal_Animals_File_S2_LegacyDatabase.pdf]

Suborder	Genus	Species	Latitute	Longitude	Ragion/Country	
Kolpophorae	Cassiopea	spp.	25.11640724	-80.46000201	Buttonwood Sound, Upper Keys, Florida, USA	[1]
Kolpophorae	Cassiopea	spp.	24.5589491	-81.80574846	Enclosure, Lower Keys, Florida, USA	[1]
Kolpophorae	Cassiopea	spp.	25.07677696	-80.4638511	Rock Harbor, Upper Keys, Florida, USA	[1]
Kolpophorae	Cassiopea	spp.	24.59307642	-81.79673663	SFUWO Drop, Lower Keys, Florida, USA	[1]
Kolpophorae	Cassiopea	spp.	24.55240169	-81.77724342	SFUWOS FAA Tower, Lower Keys, Florida, USA	[1]
Kolpophorae	Cassiopea	spp.	24.54777517	-81.78398638	SFUWOS Finger Pier, Lower Keys, Florida, USA	[1]
Kolpophorae	Cassiopea	ornata	13.46143056	144.6837333	Cabras Marina, Guam, USA	[2]
Kolpophorae	Cassiopea	spp.	13.25436667	144.6804694	Cocos Lagoon, Guam, USA	[2]
Kolpophorae	Cassiopea	ornata	13.46397222	144.655575	Outhouse Beach, Guam, USA	[2]
Kolpophorae	Cassiopea	ornata	13.46477222	144.6870472	Piti Channel, Guam, USA	[2]
Kolpophorae	Cassiopea	spp.	7.263007157	134.4159245	Milky Way Lake 1, Palau	[3]
Kolpophorae	Cassiopea	spp.	7.266237342	134.4368349	Milky Way Lake 2, Palau	[3]
Kolpophorae	Cassiopea	spp.	7.323495035	134.5088762	NGE Lake 1 Lagoon, Palau	[3]
Kolpophorae	Cassiopea	spp.	7.32159746	134.5062306	NGE Lake 2, Palau	[3]
Kolpophorae	Cassiopea	spp.	7.257846536	134.3825513	Ongael Lake, Koror State, Palau	[3]
Kolpophorae	Cassiopea	spp.	7.162755419	134.3759997	South Cassiopea Lake, Palau	[3]
Kolpophorae	Cassiopea	spp.	7.2982264	134.448364	T Lake, Palau	[3]
Kolpophorae	Cassiopea	frondosa	26.81852737	-177.8040565	FAO 6, Northwest Pacific Ocean	[4]
Kolpophorae	Cassiopea	andromeda	20.2758537	38.77549472	Subarea 51.1, Western Indian Ocean	[4]
Kolpophorae	Cassiopea	andromeda	31.18472771	33.79277222	El Arish, Egypt	[5]
Kolpophorae	Cassiopea	andromeda	34.12421097	35.46344225	Lenanese waters	[5]
Kolpophorae	Cassiopea	andromeda	24.6	81.65	Key West, Florida, USA	[6]
Kolpophorae	Cassiopea	frondosa	24.6	81.65	Key West, Florida, USA	[6]
Kolpophorae	Cassiopea	ornata	7.34	134.47	Koror, Palau	[6]
Kolpophorae	Cassiopea	xamanchana	17.95005149	-76.87073033	Port Henderson, St Catherine, Jamaica	[7]
Kolpophorae	Cassiopea	sp.	-10.99226172	142.1101384	Crab Island, Australia	[8]
Kolpophorae	Cassiopea	sp.	-26.8273881	153.1131678	Lake Magellan, Australia	[8]
Kolpophorae	Cassiopea	sp.	-14.66518283	145.4637696	Lizard Island, Australia	[8]
Kolpophorae	Cassiopea	sp.	-16.65748585	145.9906619	Vlasoff Cay, Australia	[8]
Kolpophorae	Cassiopea	sp.	-6.151648855	39.17635632	Zanzibar harbour, Tanzania	[9]
Kolpophorae	Cassiopea	sp.	-6.174807997	39.16663451	Zanzibar harbour, Tanzania	[9]
Kolpophorae	Cassiopea	xamanchana	19.89608679	-159.4903013	Hawaii	[10]
Kolpophorae	Cassiopea	xamanchana	21.11261188	-86.80028918	Nichupté lagoon, Quintana Roo, Mexico	[11]
Kolpophorae	Cassiopea	andromeda	36.18684852	36.71835476	Iskenderun Iron Steel Factory, Turkey	[12]
Kolpophorae	Cassiopea	andromeda	1.466344796	104.8485966	Singapore	[13]
Kolpophorae	Cassiopea	sp.	1.466344796	104.8485966	Singapore	[13]
Kolpophorae	Cassiopea	andromeda	13.55416667	38.12083333	Cala, Palermo, Italy	[14]
Kolpophorae	Cassiopea	frondosa	9.22349	82.21972222	Bocas del Toro, Panama	[15]
Kolpophorae	Cassiopea	xamanchana cf.	9.22349	82.21972222	Bocas del Toro, Panama	[15]
Kolpophorae	Cassiopea	andromeda	24.98334167	11.55	Isla San José, Panama	[15]
Kolpophorae	Cassiopea	xamanchana	32.88974159	-64.62087148	Bermuda	[16]
Kolpophorae	Cassiopea	andromeda	-9.472134396	147.1411117	Papua New Guinea	[16]
Kolpophorae	Cassiopea	andromeda	35.948264	14.423719	Salina Nature Reserve, Malta	[17]
Kolpophorae	Cassiopea	andromeda	35.947889	14.4239	Salina Nature Reserve, Malta	[17]
Kolpophorae	Cassiopea	sp.	27.303	-82.57535786	Lido Key, Florida Keys, USA	[18]
Kolpophorae	Cassiopea	sp.	24.52828816	-80.81391421	Long Key, Florida Keys, USA	[18]
Kolpophorae	Cassiopea	xamanchana	24.76615197	-80.95233402	Grassy Key, Florida Keys, USA	[19]
Kolpophorae	Cassiopea	xamanchana	25.31459259	-80.28442258	Key Largo, Florida Keys, USA	[19]
Kolpophorae	Cassiopea	xamanchana	27.53599983	-81.39367727	Florida Keys, USA	[20]
Kolpophorae	Cassiopea	xamanchana	-29.97500356	-50.12671062	Imbé, Rio Grande do Sul, Brazil	[20]
Kolpophorae	Cassiopea	mayeri	13.86713817	120.6259172	Calatagan, Luzon Island, Philippines	[21]
Kolpophorae	Cassiopea	culionensis	10.285649	124.000681	Lapu-Lapu, Cebu, Philippines	[21]
Kolpophorae	Cassiopea	mayeri	10.285967	124.00075	Lapu-Lapu, Cebu, Philippines	[21]
Kolpophorae	Cassiopea	mayeri	24.33759002	123.818171	Okinawa, Japan	[21]
Kolpophorae	Cassiopea	andromeda	-17.89632489	178.0381055	Dravuni, Fiji	[22]
Kolpophorae	Cassiopea	andromeda	27.28322883	33.81428616	El Ghardaqa, Egypt	[22]
Kolpophorae	Cassiopea	andromeda	-6.543129686	144.6744646	Emona, Papua New Guinea	[22]
Kolpophorae	Cassiopea	andromeda	21.28273732	-157.8392849	Hilton lagoon, Waikiki, Hawaii, USA	[22]
Kolpophorae	Cassiopea	andromeda	2.152740705	118.5271782	Kakaban, Kalimantan, Indonesia	[22]
Kolpophorae	Cassiopea	frondosa	25.09312777	-80.455916	Key Largo, Florida Keys, USA	[22]
Kolpophorae	Cassiopea	xamanchana	25.09312777	-80.455916	Key Largo, Florida Keys, USA	[22]
Kolpophorae	Cassiopea	andromeda	21.07032304	-156.9732757	Moloka'i, Hawaii, USA	[22]
Kolpophorae	Cassiopea	andromeda	7.431068774	134.6996476	Palau	[22]
Kolpophorae	Cassiopea	andromeda	-0.579904731	153.8740672	Papua New Guinea	[22]
Kolpophorae	Cassiopea	andromeda	-16.48030306	145.4632791	Port Douglas, Queensland, Australia	[22]
Kolpophorae	Cassiopea	xamanchana	32.35529417	-64.65717739	Richardson Bay, Bermuda	[22]
Kolpophorae	Cassiopea	frondosa	9.586456634	-78.78998751	San Blas Islands, Panama	[22]
Kolpophorae	Cassiopea	xamanchana	32.26895814	-64.80391971	Walsingham Pond, Bermuda	[22]
Kolpophorae	Cassiopea	andromeda	21.69260978	-157.9642887	windward O'ahu, Hawaii, USA	[22]
Kolpophorae	Cassiopea	andromeda	21.46017315	-157.8384818	windward O'ahu, Hawaii, USA	[22]
Kolpophorae	Cassiopea	andromeda	21.39152175	-157.7204654	windward O'ahu, Hawaii, USA	[22]
Kolpophorae	Cassiopea	andromeda	21.52139932	-157.8373663	windward O'ahu, Hawaii, USA	[22]
Kolpophorae	Cassiopea	xamanchana	24.65230756	-81.37485037	Big Pine Key, Florida Keys, USA	[23]
Kolpophorae	Cassiopea	xamanchana	27.32062639	-82.57060233	Coon Key, Florida Keys, USA	[23]
Kolpophorae	Cassiopea	xamanchana	26.94116437	-80.07235672	Dubois Park in Jupiter, Florida Keys, USA	[23]
Kolpophorae	Cassiopea	andromeda	8.572	81.1942	Inner Harbour, Trincomalee, Sri Lanka	[24]
Kolpophorae	Cassiopea	andromeda	9.6887	79.9959	Kaakai Theevu, Jaffna, Sri Lanka	[24]
Kolpophorae	Cassiopea	andromeda	9.6427	79.9905	Pannei, Jaffna, Sri Lanka	[24]
Kolpophorae	Cassiopea	xamanchana	9.350929301	-82.33489978	Bocas del Toro, Panama	[25]
Kolpophorae	Cassiopea	andromeda	-17.49278867	-149.8975596	Tiahura, Moorea, French Polynesia	[25]
Kolpophorae	Cassiopea	ndrosia	-34.52654293	150.8647007	Lake Illawarra, New South Wales, Australia	[26]
Kolpophorae	Cassiopea	maremetens	-32.19982749	152.5108548	Pipers Creek, New South Wales, Australia	[26]
Kolpophorae	Cassiopea	maremetens	-32.19563354	152.4988245	Wallis Lake, New South Wales, Australia	[26]
Kolpophorae	Cassiopea	ndrosia	-20.04979841	148.8833118	Whitsunday Passage, Queensland, Australia	[26]
Kolpophorae	Cassiopea	xamanchana	7.326450884	134.5085702	Big Jellyfish Lake, island of Koror, Palau	[27]

Kolpophorae	Cassiopea	frondosa	17.97666576	-67.05863942	La Parguera, Puerto Rico	[27]
Kolpophorae	Cassiopea	sp.	38.1213796	13.36833832	Cala, port of Palermo, Italy	[28]
Kolpophorae	Cassiopea	maremetens	-26.8273115	153.1131034	Lake Magellan, Sunshine Coast, Queensland, Austr	[29]
Kolpophorae	Cassiopea	andromeda	29.4017684	52.58081202	Naybandbay, Bushehr, Iran	[30]
Kolpophorae	Cassiopea	xamanchana	24.8263	-80.8139	Keys Marine Laboratory, Layton, Florida, USA	[31]
Kolpophorae	Cassiopea	sp.	29.45	34.9667	Northern Gulf of Aqaba (Red Sea), Jordan	[32]
Kolpophorae	Cassiopea	sp.	29.45	34.96666667	Northern Gulf of Aqaba (Red Sea), Jordan	[33]
Kolpophorae	Cassiopea	andromeda	36.5516717	29.11499951	Ölüdeniz Lagoon, Turkey	[34]
Kolpophorae	Cassiopea	andromeda	36.5516717	29.11499951	Ölüdeniz Lagoon, Turkey	[34]
Kolpophorae	Cassiopea	sp.	22.44140848	69.0664388	Adatra, Gujarat, India	[35]
Kolpophorae	Cassiopea	andromeda	11.54505273	92.67942594	Andaman Islands, India	[35]
Kolpophorae	Cassiopea	andromeda	22.40205505	69.20084568	Arambhada coast, gulf of Kutch, Gujarat, India	[35]
Kolpophorae	Cassiopea	andromeda var maldiv	22.42051639	69.20893651	Armara, gulf of Kutch, Gujarat, India	[35]
Kolpophorae	Cassiopea	andromeda	12.97772582	80.2715167	Chennai (Madras), Tamil Nadu, India	[35]
Kolpophorae	Cassiopea	andromeda var maldiv	9.28522557	79.20981591	Krusadai Island, Gulf of Mannar, India	[35]
Kolpophorae	Cassiopea	andromeda	22.49620463	69.71071486	Narara, gulf of Kutch, Gujarat, India	[35]
Kolpophorae	Cassiopea	xamanchana	25.50107746	-76.63394317	Girl's Bank, Harbour Island, Bahamas	[36]
Kolpophorae	Cassiopea	andromeda	26.10194025	34.28369958	Qusier (Red Sea), Egypt	[36]
Kolpophorae	Cassiopea	andromeda	38.12161071	13.36907458	Cala, port of Palermo, Italy	[37]
Kolpophorae	Cassiopea	andromeda	35.89850278	14.50586944	Marsamxett Harbour, Malta	[38]
Kolpophorae	Cassiopea	sp.	-22.83787741	-41.98810959	Cabo Frio, Brazil	[39]
Kolpophorae	Cassiopea	sp.	-9.54708211	-35.62164404	Meirim River, Maceió, Alagoas, Brazil	[40]
Kolpophorae	Cassiopea	spp.	26.36472222	77.02361111	Abaco Island, Bahama	[41]
Kolpophorae	Cassiopea	spp.	26.41762729	-77.16640925	Abaco Island, Bahama	[42]
Kolpophorae	Cassiopea	spp.	26.40435007	-77.14573843	Jungle Creek, Abaco Island, Bahamas	[43]
Kolpophorae	Cassiopea	xamanchana	27.99454391	-77.52872302	Abaco Island, Bahama	[44]
Kolpophorae	Cassiopea	ornata	7.584540711	134.5624124	Palau	[45]
Kolpophorae	Cassiopea	ornata	15.21146783	145.745401	Saipan	[45]
Kolpophorae	Cassiopea	ornata	9.879536936	138.0126757	Yap	[45]
Kolpophorae	Cassiopea	andromeda	16.28248912	114.3009022	South China Sea	[46]
Kolpophorae	Cassiopea	sp	17.58869142	-61.82210654	Codrington Lagoon, Antigua and Barbuda	[47]
Dactyliophorae	Catostylus	mosaicus	-45.96468148	-170.0102542	Pacific Ocean:Southwest Pacific (FAO 81)	[4]
Dactyliophorae	Catostylus	mosaicus	1.586173727	162.6683576	Pacific Ocean:Western Central Pacific (FAO71)	[4]
Dactyliophorae	Catostylus	mosaicus	-33.99	151.18	Botany Bay, Australia, New South Wales, Australia	[6]
Dactyliophorae	Catostylus	perezi	22.62820536	69.78575634	Gulf of Kutch, India	[48]
Dactyliophorae	Catostylus	sp.	8.124934617	123.9156414	Panguil Bay, Philippines	[49]
Dactyliophorae	Catostylus	mosaicus	-33.99985767	151.1833655	Botany Bay, New South Wales, Australia	[50]
Dactyliophorae	Catostylus	mosaicus	-34.49984083	150.8332904	Lake Illawarra, New South Wales, Australia	[50]
Dactyliophorae	Catostylus	mosaicus	-32.12657169	152.3699569	Smiths Lake, New South Wales, Australia	[50]
Dactyliophorae	Catostylus	sp.	1.466344796	104.8485966	Singapore	[13]
Dactyliophorae	Catostylus	tagi	38.99746417	-5.538124902	Guadiana river estuary, Spain	[52]
Dactyliophorae	Catostylus	sp. 1	24.17646333	-110.3157806	Bahía Jiquilisco, El Salvador	[15]
Dactyliophorae	Catostylus	sp. 1	13.15803	88.0678	Bocana del Esterón, El Salvador	[15]
Dactyliophorae	Catostylus	sp. 2	9.23620983	-83.86822983	Dominical, Coasta Rica	[15]
Dactyliophorae	Catostylus	sp. 1	13.17284	-87.88125	Golfo de Fonseca, El Salvador	[15]
Dactyliophorae	Catostylus	sp. 1	13.17284	-87.88125	Golfo de Fonseca, Nicaragua	[15]
Dactyliophorae	Catostylus	sp. 1	12.16649983	-86.79442	Puerto Sandino, Nicaragua	[15]
Dactyliophorae	Catostylus	townsendi	3.173222	101.1905833	Sg Janggut, Malaysia	[15]
Dactyliophorae	Catostylus	townsendi	-7.21613611	112.7471944	Surabaya, Indonesia	[15]
Dactyliophorae	Catostylus	townsendi	-7.259266962	112.7487301	Surabaya, Indonesia	[53]
Dactyliophorae	Catostylus	mosaicus	-26.67488736	153.1176642	Mooloolaba, Queensland, Australia	[16]
Dactyliophorae	Catostylus	mosaicus	-33.96577658	151.1831616	Botany Bay, New South Wales, Australia	[54]
Dactyliophorae	Catostylus	mosaicus	-36.016667	150.1167096	Coila Lake, NSW, New South Wales, Australia	[54]
Dactyliophorae	Catostylus	mosaicus	-37.866667	147.983333	Gippsland Lakes, Victoria, Australia	[54]
Dactyliophorae	Catostylus	mosaicus	-34.51574701	150.8336766	Lake Illawarra, New South Wales, Australia	[54]
Dactyliophorae	Catostylus	mosaicus	-26.6824511	153.1332474	Mooloolaba, Queensland, Australia	[54]
Dactyliophorae	Catostylus	mosaicus	-38.666667	146.683333	Port Albert, Victoria, Australia	[54]
Dactyliophorae	Catostylus	mosaicus	-38.09935801	144.8831616	Port Philip, Victoria, Australia	[54]
Dactyliophorae	Catostylus	mosaicus	-32.36579644	152.4996566	Smiths Lake, New South Wales, Australia	[54]
Dactyliophorae	Catostylus	mosaicus	-41.21595616	146.9331616	Tamar Estuary, Tasmania	[54]
Dactyliophorae	Catostylus	townsendi	21.39401437	111.198803	Maoming, China	[51]
Dactyliophorae	Catostylus	mosaicus	13.59149687	80.19351727	Pulicat lagoon, India	[55]
Dactyliophorae	Catostylus	perezi	24.78686149	67.02248927	Clifton, Karachi Coast, Pakistan	[56]
Dactyliophorae	Catostylus	perezi	24.79713918	66.96759656	Manora, Maharashtra, India	[56]
Dactyliophorae	Catostylus	perezi	25.44735563	66.56642274	Damb Bandar, Balochistan province, Pakistan	[57]
Dactyliophorae	Catostylus	mosaicus	-34.51737919	150.8451395	Lake Illawara, New South Wales, Australia	[22]
Dactyliophorae	Catostylus	mosaicus	-38.13981627	144.3664683	Port Philip, Victoria, Australia	[58]
Dactyliophorae	Catostylus	tagi	38.76682753	-9.085403894	Tagus estuary (near the Oceanário de Lisboa), Port	[59]
Dactyliophorae	Catostylus	mosaicus	5.46975	100.2013333	Penang National Park, Penang, Malaysia	[60]
Dactyliophorae	Catostylus	mosaicus	-23.24481625	150.3537914	Fitzroy River, Australia	[61]
Dactyliophorae	Catostylus	townsendi	4.264351273	117.898509	Tawau, Sabah, Malaysia	[62]
Dactyliophorae	Catostylus	ouwensi	-4.182749517	137.9812357	Western New Guinea	[63]
Dactyliophorae	Catostylus	tagi	38.46919289	-8.850122032	Sado river, Portugal	[64]
Dactyliophorae	Catostylus	sp.	1.466344796	104.8485966	Singapore	[65]
Dactyliophorae	Catostylus	tagi	38.47943262	-8.842315319	Sado estuary, Portugal	[66]
Dactyliophorae	Catostylus	tagi	38.68054931	-9.22910433	Tagus estuary, Portugal	[66]
Dactyliophorae	Catostylus	mosaicus	-34.54168556	150.8575579	Lake Illawara, New South Wales, Australia	[67]
Dactyliophorae	Catostylus	mosaicus	-35.68480115	150.1797578	Batemans Bay, New South Wales, Australia	[68]
Dactyliophorae	Catostylus	mosaicus	-33.98568684	151.1760799	Botany Bay, New South Wales, Australia	[68]
Dactyliophorae	Catostylus	mosaicus	-34.51455031	150.8444523	Lake Illawara, New South Wales, Australia	[68]
Dactyliophorae	Catostylus	mosaicus	-29.50489833	153.2964763	Lake Wooloweyah, New South Wales, Australia	[68]
Dactyliophorae	Catostylus	mosaicus	-32.64046956	152.1139431	Port Stephens, New South Wales, Australia	[68]
Dactyliophorae	Catostylus	mosaicus	-32.25244341	152.486352	Wallis Lake, New South Wales, Australia	[68]
Dactyliophorae	Catostylus	mosaicus	-32.36579644	152.4996566	Smiths Lake, New South Wales, Australia	[69]
Dactyliophorae	Catostylus	perezi	20.92202778	70.33330556	Veraval coast of Gujarat, India	[70]
Dactyliophorae	Catostylus	townsendi	5.999988827	102.4267222	Pantai Melawi, Kelantan, Malaysia	[71]

Dactyliophorae	Catostylus	tagi	39.92235903	-5.123297284	Tagus River, Spain	[72]
Dactyliophorae	Catostylus	mosaicus	3.728291261	102.1979729	Malaysia	[73]
Kolpophorae	Cotylorhiza	tuberculata	37.44218588	3.784939506	Mediterranean Sea:Western Mediterranean, Divisi	[4]
Kolpophorae	Cotylorhiza	tuberculata	37.77427086	-0.788933021	Mar Menor Lagoon, Spain	[74]
Kolpophorae	Cotylorhiza	tuberculata	34.12421097	35.46344225	Lebanese waters	[5]
Kolpophorae	Cotylorhiza	tuberculata	37.1122939	28.1886122	Güllük Gulf, Gulf of Gökova	[75]
Kolpophorae	Cotylorhiza	tuberculata	32.44404153	14.88408144	Al Dafiniyah, Libya	[76]
Kolpophorae	Cotylorhiza	tuberculata	39.81230388	3.224418618	Alcúdia bay, Spain	[77]
Kolpophorae	Cotylorhiza	tuberculata	45.50017294	13.5000751	Gulf of Trieste, Slovenia	[78]
Kolpophorae	Cotylorhiza	tuberculata	37.70017825	-0.833419133	Mar Menor Lagoon, Spain	[78]
Kolpophorae	Cotylorhiza	tuberculata	42.76687146	17.3500751	Mljet Lake (Adriatic Sea), Croatia	[78]
Kolpophorae	Cotylorhiza	tuberculata	38.70833333	13.19527778	Ustica Island (Tyrrhenian Sea), Italy	[78]
Kolpophorae	Cotylorhiza	tuberculata	38.68358453	20.69987125	Vlicho Bay (Ionian Sea), Island of Lefkada, Greece	[78]
Kolpophorae	Cotylorhiza	tuberculata	37.7333333	-0.78333333	Mar Menor Lagoon, Spain	[79]
Kolpophorae	Cotylorhiza	tuberculata	37.77134544	-0.784031451	Mar Menor Lagoon, Spain	[80]
Kolpophorae	Cotylorhiza	erythraea	32.559	34.905	Maagan Michael, Israel	[81]
Kolpophorae	Cotylorhiza	erythraea	32.407	34.864	Michmoret, Israel	[81]
Kolpophorae	Cotylorhiza	erythraea	32.492	34.882	Sdot Yam, Israel	[81]
Kolpophorae	Cotylorhiza	erythraea	32.073	34.757	Tel Aviv, Israel	[81]
Kolpophorae	Cotylorhiza	tuberculata	40.8741	29.0899	Sea of Marmar	[82]
Kolpophorae	Cotylorhiza	tuberculata	37.1122939	28.1886122	Güllük Gulf, Gulf of Gökova	[83]
Kolpophorae	Cotylorhiza	tuberculata	45.71074587	13.59012606	Gulf of Trieste, Slovenia	[59]
Kolpophorae	Cotylorhiza	tuberculata	37.5616239	-1.013621562	Algerian Basin, Murcia, Spain	[84]
Kolpophorae	Cotylorhiza	tuberculata	40.20194444	0.2854	Balearic Sea, Castellón, Spain	[84]
Kolpophorae	Cotylorhiza	tuberculata	0.01752778	37.56140278	Balearic Sea, Castellón, Spain	[84]
Kolpophorae	Cotylorhiza	tuberculata	39.92343986	3.944365113	Balearic Sea, Minorca, Spain	[84]
Kolpophorae	Cotylorhiza	tuberculata	39.17771111	-0.20475556	Balearic Sea, Valencia, Spain	[84]
Kolpophorae	Cotylorhiza	tuberculata	38.68647521	20.70498938	Bay of Vlyho (Ionian Sea), Greece	[84]
Kolpophorae	Cotylorhiza	tuberculata	39.09337757	9.4856193	Capo Carbonara, Sardinia, Italy	[84]
Kolpophorae	Cotylorhiza	tuberculata	39.09823043	9.552365484	Capo Carbonara, Sardinia, Italy	[84]
Kolpophorae	Cotylorhiza	tuberculata	40.03942706	15.29045255	Palinuro, Campania, Italy	[84]
Kolpophorae	Cotylorhiza	tuberculata	40.81902375	14.12244974	Pozzuoli, Campania, Italy	[84]
Kolpophorae	Cotylorhiza	tuberculata	45.52692089	13.56775103	Bay of Piran (Northern Adriatic Sea), Slovenia	[85]
Kolpophorae	Cotylorhiza	tuberculata	40.95460146	17.00655167	Apulia coasts, Italy	[86]
Kolpophorae	Cotylorhiza	tuberculata	40.4724005	16.93841949	Castellaneta Marina and Pulsano (Ionian Sea), Italy	[87]
Kolpophorae	Cotylorhiza	tuberculata	40.464985	17.19758977	Salento Peninsula coastline (Ionian Sea), Italy	[88]
Kolpophorae	Cotylorhiza	tuberculata	40.22430717	18.47220469	South Adriatic Seas	[88]
Kolpophorae	Cotylorhiza	tuberculata	41.38416097	2.204771455	Barcelona	[89]
Kolpophorae	Cotylorhiza	tuberculata	36.9609	10.4846	Tunis Gulf	[90]
Dactyliophorae	Eupilema	inexpectata	-33.95776146	25.62081079	Port Elizabeth, South Africa	[91]
Dactyliophorae	Eupilema	inexpectata	-34.00375002	23.45981147	Keurbooms, South Africa	[92]
Dactyliophorae	Eupilema	inexpectata	-33.86443853	25.63218244	Port Elizabeth, South Africa	[92]
Dactyliophorae	Eupilema	inexpectata	-34.04584282	18.35444612	Hout Bay Beach, Hout Bay, Cape Town, South Afric	[93]
Dactyliophorae	Lobonema	smithii sp. 1	25.14313	-112.14509	Agonales, Mexico	[15]
Dactyliophorae	Lobonema	smithii sp. 1	24.17646333	-110.3157806	Bahía de la Paz, Mexico	[15]
Dactyliophorae	Lobonema	smithii sp. 1	25.18893	-112.12913	Canal Principal, Mexico	[15]
Dactyliophorae	Lobonema	smithii sp.2	13.15924	-88.0435	El Coquito, El Salvador	[15]
Dactyliophorae	Lobonema	smithii sp.4	13.17284	-87.88125	Golfo de Fonseca, El Salvador	[15]
Dactyliophorae	Lobonema	smithii	8.18341694	98.48349639	Ko Panak, Thailand	[15]
Dactyliophorae	Lobonema	smithii	3.17322222	101.1905833	Sg Janggut, Malaysia	[15]
Dactyliophorae	Lobonema	smithii sp.3	8.00048889	-79.49161111	Tocumen, Panama	[15]
Dactyliophorae	Lobonema	mayeri	10.8482231	119.3647238	Malampaya Sound, Taytay, Philippines	[95]
Dactyliophorae	Lobonema	smithii	-1.237145542	116.8564511	Balikpepan and Kotabalu (East Kalimantan, Makass	[94]
Dactyliophorae	Lobonema	smithii	-2.176797121	106.0658114	Bangka Island (South Sumatra, Java Sea)	[94]
Dactyliophorae	Lobonema	smithii	11.96633834	109.2018375	Cam Ranh (South China Sea), Vietnam	[94]
Dactyliophorae	Lobonema	smithii	11.36997549	124.6785164	Carigara Bay, Philippines	[94]
Dactyliophorae	Lobonema	smithii	5.075252162	99.65547819	Ipoh and Kuala Lumpur, Strait of Malacca	[94]
Dactyliophorae	Lobonema	smithii	1.805758253	111.107308	Kabong (South China Sea), Malaysia	[94]
Dactyliophorae	Lobonema	smithii	1.55705798	110.3512249	Kuching (South China Sea), Malaysia	[94]
Dactyliophorae	Lobonema	smithii	10.85046891	119.3646189	Malampaya Sound, Philippines	[94]
Dactyliophorae	Lobonema	smithii	3.615275986	98.6795113	Medan (North Sumatra)	[94]
Dactyliophorae	Lobonema	smithii	4.231722995	100.5580664	Pangkor, Strait of Malacca	[94]
Dactyliophorae	Lobonema	smithii	5.420030133	100.3250237	Penang, Strait of Malacca	[94]
Dactyliophorae	Lobonema	smithii	10.30246158	103.9855666	Phu Quoc Island, Gulf of Thailand	[94]
Dactyliophorae	Lobonema	smithii	10.44157745	119.1284152	Port Barton (Palawan), Philippines	[94]
Dactyliophorae	Lobonema	smithii	9.952449918	98.6058334	Ranong, Andaman Sea	[94]
Dactyliophorae	Lobonema	smithii	12.68644166	101.2808323	Rayong, Gulf of Thailand	[94]
Dactyliophorae	Lobonema	smithii	13.54400496	100.272174	Samut Sakhon, Gulf of Thailand	[94]
Dactyliophorae	Lobonema	smithii	13.92619752	123.2204567	San Miguel Bay, Philippines	[94]
Dactyliophorae	Lobonema	smithii	1.808565006	109.7741533	Sematan (South China Sea), Malaysia	[94]
Dactyliophorae	Lobonema	smithii	20.15520741	92.87051926	Sittwe (Arakan, Bay of Bengal)	[94]
Dactyliophorae	Lobonema	smithii	4.028576755	101.0160336	Telok Anson, Strait of Malacca	[94]
Dactyliophorae	Lobonema	smithii	-6.895495909	112.0402914	Tuban (East Java, Java Sea)	[94]
Dactyliophorae	Lobonema	smithii	13.28402769	100.9141765	Bangsean Beach, Chonburi	[96]
Dactyliophorae	Lobonema	smithii	13.27002836	100.9220522	Wonnapa Beach, Chonburi Province	[96]
Dactyliophorae	Lychnorhiza	lucerna	-20.64491396	-46.13969763	Atlantic Ocean: Southwest Atlantic, Division 41.2.1	[4]
Dactyliophorae	Lychnorhiza	lucerna	-0.783342096	-48.01104657	Mojuim estuary, São Caetano de Odivelas, Pará, Br	[97]
Dactyliophorae	Lychnorhiza	lucerna	-36.35	-56.71	San Clemente del Tuyu, Argentina	[6]
Dactyliophorae	Lychnorhiza	lucerna	10.98494337	-74.30442295	Isla de Salamanca (Magdalena)	[98]
Dactyliophorae	Lychnorhiza	lucerna	-35.93555	-56.98562222	Bahía Saborombón, Argentina	[15]
Dactyliophorae	Lychnorhiza	sp. 1	9.23620983	-83.86822983	Dominical, Coasta Rica	[15]
Dactyliophorae	Lychnorhiza	lucerna	14.02275972	-83.40319972	Estero Guililanding-Bilwi Tigni, Nicaragua	[15]
Dactyliophorae	Lychnorhiza	sp. 2	9.62485	-82.61739	Gandoca, Costa Rico	[15]
Dactyliophorae	Lychnorhiza	sp. 1	8.5637	-79.82227	Gorgona, Panama	[15]
Dactyliophorae	Lychnorhiza	lucerna	14.08112	-83.39164983	Guililanding, Nicaragua	[15]
Dactyliophorae	Lychnorhiza	sp. 3	11.10995806	-63.97120111	Isla Margarita, Venezuela	[15]

Dactyliophorae	Lychnorhiza	sp. 1	13.15828	-87.98991	Las Tunas, El Salvador	[15]
Dactyliophorae	Lychnorhiza	sp. 1	11.66802983	-86.57469	Masachapa, Nicaragua	[15]
Dactyliophorae	Lychnorhiza	sp. 1	15.66193206	-96.49210453	Salina Cruz, Mexico	[15]
Dactyliophorae	Lychnorhiza	sp. 1	8.00048889	-79.49161111	Tocumen, Panama	[15]
Dactyliophorae	Lychnorhiza	lucerna	14.10191972	-83.32129983	Tuapí, Nicaragua	[15]
Dactyliophorae	Lychnorhiza	malayensis	-7.259266962	112.7487301	Surabaya, Indonesia	[53]
Dactyliophorae	Lychnorhiza	lucerna	-25.72830495	-48.38424096	Currais Islands, Paraná, southern Brazil	[99]
Dactyliophorae	Lychnorhiza	lucerna	-25.88117465	-48.56788602	shoreline of city of Guaratuba, Brazil	[99]
Dactyliophorae	Lychnorhiza	lucerna	-25.88117465	-48.56788602	shoreline of city of Guaratuba, Brazil	[100]
Dactyliophorae	Lychnorhiza	lucerna	-27.49970497	-48.53335476	North Bay, Santa Catarina, southern Brazil	[101]
Dactyliophorae	Lychnorhiza	sp.	8.308334267	98.46072459	Ao Phang Nga, Thailand	[102]
Dactyliophorae	Lychnorhiza	sp.	8.31476	98.27028	Nai Rai Village, Thailand	[102]
Dactyliophorae	Lychnorhiza	sp.	8.85994	98.27201	Nam Kem Village Phanggah, thailand	[102]
Dactyliophorae	Lychnorhiza	lucerna	-24.99975689	-47.99994636	São Paulo State, southeastern Brazil)	[103]
Dactyliophorae	Lychnorhiza	lucerna	-3.719107131	-38.50507132	Brazil, Ceará State, Fortaleza, Meireles beach	[104]
Dactyliophorae	Lychnorhiza	lucerna	-25.0089256	-47.93533459	Cananéia Estuary, South Brazil Bight	[105]
Dactyliophorae	Lychnorhiza	lucerna	-25.48631041	-48.43314578	Paranaguá Estuary, South Brazil Bight	[105]
Dactyliophorae	Lychnorhiza	lucerna	-23.80912142	-45.39738683	São Sebastião Channel, South Brazil Bight	[105]
Dactyliophorae	Lychnorhiza	lucerna	-25.0664626	-47.86672035	Cananéia Estuary, Southeast Brazil,	[106]
Dactyliophorae	Lychnorhiza	malayensis	9.421921571	76.67342346	Kerala state, India	[107]
Dactyliophorae	Lychnorhiza	lucerna	-2.537848914	-44.17154373	Cururuca's River (São José de Ribamar municipe)	[108]
Dactyliophorae	Lychnorhiza	lucerna	-36.15088988	-56.87337387	Argentinean coast of the Rio de la Plata estuary	[109]
Dactyliophorae	Lychnorhiza	lucerna	-25.00929014	-47.92978251	Cananéia region, Brazil	[110]
Dactyliophorae	Lychnorhiza	lucerna	-35.0157218	-56.72577954	Rıo de la Plata estuary, Argentina	[110]
Dactyliophorae	Lychnorhiza	lucerna	-35.0157218	-56.72577954	Rıo de la Plata estuary, Argentina	[110]
Dactyliophorae	Lychnorhiza	lucerna	-36.34868535	-56.7167551	San Clemente del Tuyú, Argentina	[39]
Dactyliophorae	Lychnorhiza	lucerna	-4.836659878	-37.24828742	Brazil, Rio Grande do Norte, Tibaú Beach	[111]
Dactyliophorae	Lychnorhiza	lucerna	-3.717969088	-38.48292305	Enseada do Mucuripe, Fortaleza - State of Ceará, B	[111]
Dactyliophorae	Lychnorhiza	malayensis	5.39272222	100.1619444	Balik Pulau, Penang, Malaysia	[112]
Dactyliophorae	Lychnorhiza	malayensis	2.89736111	101.3054444	Carey Island, Selangor, Malayasia	[112]
Dactyliophorae	Lychnorhiza	malayensis	6.290472	99.725389	Cenang Beach, Langkawi, Kedah, Malaysia	[112]
Dactyliophorae	Lychnorhiza	malayensis	5.31488889	100.3051389	Jerejak Island, Penang, Malaysia	[112]
Dactyliophorae	Lychnorhiza	malayensis	6.42163889	99.86694444	Kilim Geoforest Park, Langkawi, Kedah, Malaysia	[112]
Dactyliophorae	Lychnorhiza	malayensis	3.16855556	101.279	Klang Strait, Selangor, Malayasia	[112]
Dactyliophorae	Lychnorhiza	malayensis	6.238444	102.2461	Kuala Besar (Kelantan)	[112]
Dactyliophorae	Lychnorhiza	malayensis	6.13711111	102.36975	Kuala Kemasin (Kelantan)	[112]
Dactyliophorae	Lychnorhiza	malayensis	5.99611111	102.4371389	Melawi Beach (Kelantan)	[112]
Dactyliophorae	Lychnorhiza	malayensis	5.46975	100.2013333	Penang National Park, Penang, Malaysia	[112]
Dactyliophorae	Lychnorhiza	malayensis	5.27305556	100.2858611	Teluk Tempoyak, Penang, Malaysia	[112]
Dactyliophorae	Lychnorhiza	malayensis	3.16855556	101.279	Klang Strait, Selangor, Malayasia	[113]
Dactyliophorae	Lychnorhiza	malayensis	6.13711111	102.36975	Kuala Besar (Kelantan)	[113]
Dactyliophorae	Lychnorhiza	lucerna	-25.86684875	-48.60278565	Baía de Guaratuba - Guaratuba, Paraná, Brazil	[114]
Dactyliophorae	Lychnorhiza	lucerna	-37.74983032	-57.4334084	Mar Chiquita Estuary	[115]
Dactyliophorae	Pseudorhiza	haeckeli	-38.08	145.05	Port Phillip Bay, Australia	[6]
Dactyliophorae	Pseudorhiza	haeckeli	-38.13014711	144.8278974	Port Philip, Victoria	[116]
Dactyliophorae	Pseudorhiza	haeckeli	-38.10854569	144.8828139	Port Phillip Bay	[117]
Dactyliophorae	Pseudorhiza	haeckeli	-38.13981627	144.3664683	Port Philip, Victoria	[58]
Dactyliophorae	Rhizostoma	pulmo	43.4095	10.5108	Castiglioncello (CS)	[4]
Dactyliophorae	Rhizostoma	pulmo	36.9476	-6.4873	Doñana National Park (DN)	[4]
Dactyliophorae	Rhizostoma	pulmo	37.6776	0.7912	Mar Menor Lagoon, Spain	[4]
Dactyliophorae	Rhizostoma	pulmo	37.8092	0.9228	Mar Menor Lagoon, Spain	[4]
Dactyliophorae	Rhizostoma	pulmo	43.8985	10.3305	Marina di Vecchiano (MV)	[4]
Dactyliophorae	Rhizostoma	pulmo	43.7463	10.3857	Western Mediterranean, Calambrone (CA)	[4]
Dactyliophorae	Rhizostoma	pulmo	31.18472771	33.79277222	El Arish	[5]
Dactyliophorae	Rhizostoma	pulmo	34.12421097	35.46344225	Lenanese waters	[5]
Dactyliophorae	Rhizostoma	pulmo	46	37	Sea of Azov	[131]
Dactyliophorae	Rhizostoma	pulmo	40.43348228	16.88373216	Ginosa Marina (Ionian Sea), Italy	[118]
Dactyliophorae	Rhizostoma	pulmo	45.52	13.56	Gulf of Trieste, Slovenia	[6]
Dactyliophorae	Rhizostoma	pulmo	32.44404153	14.88408144	Al Dafiniyah, Libya	[76]
Dactyliophorae	Rhizostoma	pulmo	36.4692	10.7822	Tunisian coast, Benikhiar (BK)	[119]
Dactyliophorae	Rhizostoma	pulmo	37.2763	9.8731	Tunisian coast, Bizerte (BZ)	[119]
Dactyliophorae	Rhizostoma	pulmo	33.25	11.2667	Tunisian coast, El Bibane (BB)	[119]
Dactyliophorae	Rhizostoma	pulmo	37.05	11.0167	Tunisian coast, El Haouaria (HA)	[119]
Dactyliophorae	Rhizostoma	pulmo	33.8815	10.0982	Tunisian coast, Gabes (GA)	[119]
Dactyliophorae	Rhizostoma	pulmo	36.818	10.305	Tunisian coast, Halk El Oued (HW)	[119]
Dactyliophorae	Rhizostoma	pulmo	36.0303	10.509	Tunisian coast, Hergla (HE)	[119]
Dactyliophorae	Rhizostoma	pulmo	35.3944	11.0436	Tunisian coast, Salakta (SA)	[119]
Dactyliophorae	Rhizostoma	pulmo	34.7406	10.7603	Tunisian coast, Sfax (SF)	[119]
Dactyliophorae	Rhizostoma	pulmo	37.77134544	-0.784031451	Mar Menor Lagoon, Spain	[80]
Dactyliophorae	Rhizostoma	pulmo	40.31120698	2.174523989	Catalan Sea	[120]
Dactyliophorae	Rhizostoma	pulmo	37.77152546	-0.785964842	Mar Menor Lagoon, Spain	[120]
Dactyliophorae	Rhizostoma	octopus	54.1895962	7.859389306	Helgoland, German Bight, North Sea	[122]
Dactyliophorae	Rhizostoma	octopus	54.18387888	7.910589868	Helgoland, German Bight, North Sea	[121]
Dactyliophorae	Rhizostoma	pulmo	50.54988481	-2.434782525	Isle of Portland, UK	[91]
Dactyliophorae	Rhizostoma	luteum	36.72102632	-3.728308648	Marina del Este, La Herradura, southern Spain	[123]
Dactyliophorae	Rhizostoma	luteum	36.720278	-3.728333	Marina del Este, La Herradura, southern Spain	[123]
Dactyliophorae	Rhizostoma	pulmo	39.81156735	17.3354193	Gulf of Taranto (Italy, Ionian Sea)	[59]
Dactyliophorae	Rhizostoma	pulmo	45.71074587	13.59012606	Gulf of Trieste (Slovenia, Northern Adriatic Sea)	[59]
Dactyliophorae	Rhizostoma	octopus	51.67640108	4.500610662	Carmarthen Bay, Wales	[124]
Dactyliophorae	Rhizostoma	octopus	53.68032488	6.191073486	Gormanstown, Ireland	[124]
Dactyliophorae	Rhizostoma	octopus	46.16152881	-1.157371281	La Rochelle, France	[124]
Dactyliophorae	Rhizostoma	octopus	52.29279444	6.34431944	Rosslare, Ireland	[124]
Dactyliophorae	Rhizostoma	octopus	52.8766065	4.183225778	Tremadoc Bay, Wales	[124]
Dactyliophorae	Rhizostoma	pulmo	45.52692089	13.56775103	Bay of Piran (Northern Adriatic Sea, Slovenia)	[85]
Dactyliophorae	Rhizostoma	pulmo	40.4724005	16.93841949	Ionian Sea (Castellaneta Marina and Pulsano, Italy)	[87]
Dactyliophorae	Rhizostoma	pulmo	40.14790663	18.48827844	Southern Adriatic Sea (Otranto, Italy)	[87]

Dactyliophorae	Rhizostoma	pulmo	40.464985	17.19758977	Salento Peninsula coastline, Ionian	[88]
Dactyliophorae	Rhizostoma	pulmo	40.22430717	18.47220469	South Adriatic Seas	[88]
Dactyliophorae	Rhizostoma	spp.	53.29838647	-4.429838552	Anglesey	[125]
Dactyliophorae	Rhizostoma	spp.	44.41391423	-68.96340217	Belfast Bay	[125]
Dactyliophorae	Rhizostoma	spp.	51.01918252	-4.212206951	Bideford	[125]
Dactyliophorae	Rhizostoma	spp.	51.69370174	-4.398509362	Carmarthen Bay	[125]
Dactyliophorae	Rhizostoma	spp.	51.69370174	-4.398509362	Carmarthen Bay	[125]
Dactyliophorae	Rhizostoma	spp.	55.70320459	12.61457077	Copenhagen	[125]
Dactyliophorae	Rhizostoma	spp.	50.43398029	-5.301692208	Cornwall	[125]
Dactyliophorae	Rhizostoma	spp.	50.82676931	-0.270323813	Cornwall to Shoreham, West Sussex	[125]
Dactyliophorae	Rhizostoma	spp.	55.75012852	-4.932840689	Cumbrae, Clyde	[125]
Dactyliophorae	Rhizostoma	spp.	53.32405147	-6.127790585	Dublin	[125]
Dactyliophorae	Rhizostoma	spp.	53.32405147	-6.127790585	Dublin	[125]
Dactyliophorae	Rhizostoma	spp.	57.4438474	10.56878198	Frederickshaven	[125]
Dactyliophorae	Rhizostoma	spp.	57.44680329	10.53307642	Frederickshaven	[125]
Dactyliophorae	Rhizostoma	spp.	57.12565428	8.609313785	Hantsholm/Hitshals	[125]
Dactyliophorae	Rhizostoma	spp.	54.17962402	7.890597221	Helgoland	[125]
Dactyliophorae	Rhizostoma	spp.	54.17962402	7.890597221	Helgoland	[125]
Dactyliophorae	Rhizostoma	spp.	54.17962402	7.890597221	Helgoland region	[125]
Dactyliophorae	Rhizostoma	spp.	52.6773143	5.414782807	Holland	[125]
Dactyliophorae	Rhizostoma	spp.	55.72426109	-4.901577896	Hunterston, Clyde	[125]
Dactyliophorae	Rhizostoma	spp.	51.06655456	1.079860566	Hythe, Kent	[125]
Dactyliophorae	Rhizostoma	spp.	54.0460882	-4.421470307	Irish Sea	[125]
Dactyliophorae	Rhizostoma	spp.	51.45545696	0.72530931	Isle of Grain, Kent	[125]
Dactyliophorae	Rhizostoma	spp.	54.22651294	-4.498100697	Isle of Man	[125]
Dactyliophorae	Rhizostoma	spp.	56.18628938	-2.566838838	Isle of May	[125]
Dactyliophorae	Rhizostoma	spp.	56.26323185	9.150617497	Jutland	[125]
Dactyliophorae	Rhizostoma	spp.	58.15155142	7.987248739	Kristianiafjorden	[125]
Dactyliophorae	Rhizostoma	spp.	53.71788009	-3.019014521	Lancashire	[125]
Dactyliophorae	Rhizostoma	spp.	51.20450367	-3.478934035	Minehead	[125]
Dactyliophorae	Rhizostoma	spp.	57.80504485	-3.502284368	Moray Firth	[125]
Dactyliophorae	Rhizostoma	spp.	50.12247385	-5.469164801	Mounts Bay	[125]
Dactyliophorae	Rhizostoma	spp.	50.12247385	-5.469164801	Mounts Bay	[125]
Dactyliophorae	Rhizostoma	spp.	51.15062479	2.715172591	Nieuwpoortbad, Belgium	[125]
Dactyliophorae	Rhizostoma	spp.	56.05860375	-2.720572092	North Berwick	[125]
Dactyliophorae	Rhizostoma	spp.	55.53885664	-1.607880641	Northumberland	[125]
Dactyliophorae	Rhizostoma	spp.	64.36763908	9.707855363	Norwegian coast	[125]
Dactyliophorae	Rhizostoma	spp.	53.38024724	-6.057541911	Off Howth, Dublin	[125]
Dactyliophorae	Rhizostoma	spp.	54.48568697	-5.583000495	Off Strangford Lough	[125]
Dactyliophorae	Rhizostoma	spp.	51.22454445	2.910174443	Ostend, Belgium	[125]
Dactyliophorae	Rhizostoma	spp.	51.80798011	-5.264354367	Pembrokeshire	[125]
Dactyliophorae	Rhizostoma	spp.	50.3602085	-4.139941765	Plymouth	[125]
Dactyliophorae	Rhizostoma	spp.	55.29894531	-6.208458761	Rathlin Island, County Antrim	[125]
Dactyliophorae	Rhizostoma	spp.	52.25778904	-6.33835852	Rosslare harbour	[125]
Dactyliophorae	Rhizostoma	spp.	53.52589495	-6.090366784	Rush, Dublin	[125]
Dactyliophorae	Rhizostoma	spp.	54.75207497	-3.665809185	Solway Firth	[125]
Dactyliophorae	Rhizostoma	spp.	54.75207497	-3.665809185	Solway Firth	[125]
Dactyliophorae	Rhizostoma	spp.	54.75207497	-3.665809185	Solway Firth	[125]
Dactyliophorae	Rhizostoma	spp.	50.89876737	1.161730148	South coast	[125]
Dactyliophorae	Rhizostoma	spp.	50.89876737	1.161730148	South coast	[125]
Dactyliophorae	Rhizostoma	spp.	50.52839402	-3.499470151	South-west coast	[125]
Dactyliophorae	Rhizostoma	spp.	52.75299244	-5.983966499	South-west region	[125]
Dactyliophorae	Rhizostoma	spp.	52.75299244	-5.983966499	South-west region	[125]
Dactyliophorae	Rhizostoma	spp.	50.21531518	-5.471565001	St Ives	[125]
Dactyliophorae	Rhizostoma	spp.	50.15308523	-5.064912201	St Ives/Falmouth	[125]
Dactyliophorae	Rhizostoma	spp.	54.48568697	-5.583000495	Strangford Lough	[125]
Dactyliophorae	Rhizostoma	spp.	52.64274331	4.503311012	Stuifdijk/Veerhaven	[125]
Dactyliophorae	Rhizostoma	spp.	56.07388741	15.7786128	Swedish coast	[125]
Dactyliophorae	Rhizostoma	spp.	53.86795164	7.070424545	Texel, Wadden Sea	[125]
Dactyliophorae	Rhizostoma	spp.	52.87327661	-4.195281164	Tremadog Bay	[125]
Dactyliophorae	Rhizostoma	spp.	52.87327661	-4.195281164	Tremadog Bay	[125]
Dactyliophorae	Rhizostoma	spp.	39.45358555	-0.321346782	Valencia	[125]
Dactyliophorae	Rhizostoma	spp.	39.45358555	-0.321346782	Valencia	[125]
Dactyliophorae	Rhizostoma	spp.	39.45358555	-0.321346782	Valencia	[125]
Dactyliophorae	Rhizostoma	spp.	39.45358555	-0.321346782	Valencia	[125]
Dactyliophorae	Rhizostoma	spp.	51.36384166	1.019072895	Whitstable Bay, Thames estuary	[125]
Dactyliophorae	Rhizostoma	spp.	51.95424236	-7.845819198	Youghal, Cork	[125]
Dactyliophorae	Rhizostoma	pulmo	46	37	Sea of Azov	[126]
Dactyliophorae	Rhizostoma	pulmo	24.13882334	67.45287034	Keti Bandar, Thatta	[127]
Dactyliophorae	Rhizostoma	octopus	53.98411506	-10.0180592	Achill Island, Co Mayo, Ireland	[128]
Dactyliophorae	Rhizostoma	octopus	52.17815944	-6.364310186	Carnsore Point Co. Wexford, Ireland	[128]
Dactyliophorae	Rhizostoma	octopus	51.63891825	-8.628599521	Courtmacsherry Bay, Co Cork, Ireland	[128]
Dactyliophorae	Rhizostoma	octopus	52.11220853	-10.17460618	Dingle Bay, Co Kerry, Ireland	[128]
Dactyliophorae	Rhizostoma	octopus	54.65826014	-8.52835588	Donegal Bay, Co Donegal, Ireland	[128]
Dactyliophorae	Rhizostoma	octopus	52.11471242	-7.623409784	Dungarvan Co. Waterford, Ireland	[128]
Dactyliophorae	Rhizostoma	octopus	53.20256505	-9.235393541	Galway Bay, Ireland	[128]
Dactyliophorae	Rhizostoma	octopus	53.61978432	-9.759639266	Killary Harbour, Co Galway, Ireland	[128]
Dactyliophorae	Rhizostoma	octopus	53.68043827	-6.238836237	Laytown, Co Louth	[128]
Dactyliophorae	Rhizostoma	octopus	52.9410508	-9.391387602	Liscannor, Co Clare, Ireland	[128]
Dactyliophorae	Rhizostoma	octopus	52.516667	-6.016667	nil - (Muir Éireann, Ireland)	[128]
Dactyliophorae	Rhizostoma	octopus	54.27396738	-9.122092445	North Mayo, Killala Bay, Ireland	[128]
Dactyliophorae	Rhizostoma	octopus	55.44132732	-6.177378682	Rathlin Island, Co Antrim	[128]
Dactyliophorae	Rhizostoma	octopus	52.34877448	-6.359826176	Raven Point, Co. Wexford, Ireland	[128]
Dactyliophorae	Rhizostoma	octopus	54.48478949	-5.583515336	Strangford Lough, Co Down	[128]
Dactyliophorae	Rhizostoma	pulmo	45.56016998	13.73933667	Bay of Koper, Slovenia	[129]
Dactyliophorae	Rhizostoma	pulmo	45.53756285	13.61048784	Bay of Strunjan, Slovenia	[129]

Dactyliophorae	Rhizostoma	pulmo	36.86878421	10.39750347	Gulf of Tunis, Tunisia	[129]
Dactyliophorae	Rhizostoma	pulmo	37.20155077	9.867196372	Lake Bizerte, Tunisia	[129]
Dactyliophorae	Rhizostoma	pulmo	37.7718647	-0.785707381	Mar Menor Lagoon, Spain	[129]
Dactyliophorae	Rhizostoma	pulmo	41.88370168	15.74759235	Varano Lagoon, Italy	[129]
Dactyliophorae	Rhizostoma	pulmo	45.35344729	12.27085916	Venetian Lagoon, Italy	[129]
Dactyliophorae	Rhizostoma	pulmo	45.59117018	12.92597798	North Adriatic Sea	[130]
Dactyliophorae	Rhizostoma	pulmo	32.62509416	33.53961585	South Mediterranean	[130]
Dactyliophorae	Rhizostoma	pulmo	36.90539302	-1.687460924	West Mediterranean	[130]
Dactyliophorae	Rhizostoma	luteum	40.73469703	-8.938547013	Portugal	[72]
Dactyliophorae	Rhizostoma	pulmo	37.77308202	-0.786394487	Mar Menor Lagoon, Spain	[39]
Dactyliophorae	Rhizostoma	pulmo	36.9609	10.4846	Tunis Gulf, Soutwest Mediterranean	[90]
Dactyliophorae	Rhopilema	nomadica	33.34624985	31.43600003	Mediterranean Sea:Eastern Mediterranean, Divisic	[4]
Dactyliophorae	Rhopilema	esculentum	26.81852737	-177.8040565	Pacific Ocean:Northwest Pacific (FAO 61)	[4]
Dactyliophorae	Rhopilema	nomadica	36.61722308	36.17923482	Iskenderun Bay, Turkey	[131]
Dactyliophorae	Rhopilema	esculentum	5.071772449	118.3325982	Lahad Datu, Sabah	[132]
Dactyliophorae	Rhopilema	verrilli	30.20344582	-87.80783936	Alabama, USA	[133]
Dactyliophorae	Rhopilema	verilli	30.2	-88.19	Dauphin Island, AL, USA	[6]
Dactyliophorae	Rhopilema	verrilli	37.26193633	-76.38126304	York River Entrance, Virginia, USA	[134]
Dactyliophorae	Rhopilema	hispidum	-7.259266962	112.7487301	Surabaya, Indonesia	[53]
Dactyliophorae	Rhopilema	nomadica	36.05486005	14.19160344	Dwejra, Gozo, Malta	[135]
Dactyliophorae	Rhopilema	nomadica	36.00775063	14.39963156	Sikka l-Bajda, Malta	[135]
Dactyliophorae	Rhopilema	esculentum	36.44477672	120.7988118	Aoshan Bay, Qingdao, Shandong Province, China	[136]
Dactyliophorae	Rhopilema	nomadica	38.42518966	27.14314422	Izmir, Turkey	[137]
Dactyliophorae	Rhopilema	nomadica	31.8044	34.6553	Ashdod, Israel	[138]
Dactyliophorae	Rhopilema	nomadica	32.3817	34.8635	Beit Yanai, Israel	[138]
Dactyliophorae	Rhopilema	nomadica	32.7523	34.9575	Hahotrim, Haifa, Israel	[138]
Dactyliophorae	Rhopilema	nomadica	32.61836722	34.91737121	Tel Dor (30 km south of Haifa), Israel	[138]
Dactyliophorae	Rhopilema	hispidum	24.13038936	67.45308643	Keti Bandar, Sindh province, Pakistan	[57]
Dactyliophorae	Rhopilema	nomadica	36.84747222	28.27035833	Marmaris, Turkey	[139]
Dactyliophorae	Rhopilema	hispidum	13.33270721	100.9192923	Ang Sila, Thailand	[140]
Dactyliophorae	Rhopilema	hispidum	20.72148968	106.7929592	Do son, Vietnam	[140]
Dactyliophorae	Rhopilema	hispidum	1.327060896	103.443407	Kukup, Malaysia	[140]
Dactyliophorae	Rhopilema	hispidum	13.02377983	100.084941	Phetchaburi, Thailand	[140]
Dactyliophorae	Rhopilema	hispidum	7.222935463	100.6222201	Songkhla, Thailand	[140]
Dactyliophorae	Rhopilema	hispidum	13.17835094	100.9311799	Sri Racha, thailand	[140]
Dactyliophorae	Rhopilema	hispidum	19.72183344	105.8880828	Thanh Hon, Vietnam	[140]
Dactyliophorae	Rhopilema	esculentum	3.995	100.7602222	Bagan Datoh, Perak, Malayasia	[141]
Dactyliophorae	Rhopilema	hispidum	1.326960268	103.4467076	Kukup (southwest Johor), Malaysia	[141]
Dactyliophorae	Rhopilema	nomadica	36.82457316	34.63532801	Mersin, Turkey	[142]
Dactyliophorae	Rhopilema	hispidum	20.1543252	92.86773069	Sittwe, Myanmar	[143]
Dactyliophorae	Rhopilema	hispidum	-3.122592202	115.3678058	South Kalimantan, Indonesia	[143]
Dactyliophorae	Rhopilema	nomadica	32.82464308	35.02381401	Eastern Mediterranean, Haifa, Israel	[59]
Dactyliophorae	Rhopilema	nomadica	32.4063889	34.87333333	Mikhmoret, Israel	[144]
Dactyliophorae	Rhopilema	hispidum	5.46975	100.2013333	Penang National Park, Penang, Malaysia	[60]
Dactyliophorae	Rhopilema	nomadica	35.55486749	35.79222563	Lattakia, Syria	[145]
Dactyliophorae	Rhopilema	nomadica	34.09620685	35.75899097	Lebanon	[145]
Dactyliophorae	Rhopilema	esculentum	40.56123317	121.5138598	Liaodong Bay, China	[146]
Dactyliophorae	Rhopilema	nomadica	32.82485945	35.02355655	Haifa Bay, Israel	[147]
Dactyliophorae	Rhopilema	nomadica	33.98633126	35.63419148	Jounieh, Lebanon	[148]
Dactyliophorae	Rhopilema	nomadica	35.55486749	35.79222563	Lattakia, Syria	[148]
Dactyliophorae	Rhopilema	nomadica	32.08494351	34.77248375	Tel Aviv, Israel	[148]
Dactyliophorae	Rhopilema	esculentum	3.995	100.7602222	Bagan Datoh, Perak, Malayasia	[149]
Dactyliophorae	Rhopilema	hispidum	1.326960268	103.4467076	Kukup (southwest Johor), Malaysia	[149]
Dactyliophorae	Rhopilema	hispidum	1.333359766	103.4406989	Kukup, Johhor, Malaysia	[150]
Dactyliophorae	Rhopilema	sp.	12.58913072	100.9670529	Rad Island, Samae-sarn Islands, Gulf of thailand	[150]
Dactyliophorae	Rhopilema	hispidum	13.11279506	100.8089141	Sichang Island, Gulf of Thailand	[150]
Dactyliophorae	Rhopilema	esculentum	33.17864005	130.2448651	Ariake Sea, Japan	[151]
Dactyliophorae	Rhopilema	hispidum	33.17864005	130.2448651	Ariake Sea, Japan	[151]
Dactyliophorae	Rhopilema	esculentum	31.41988862	130.252831	Kaseda, Japan	[151]
Dactyliophorae	Rhopilema	sp.	-0.528944113	127.5060026	Bacan Island (Halmahera)	[94]
Dactyliophorae	Rhopilema	hispidum	-6.731219791	108.5637527	Cirebon, West Java, Indian Ocean	[94]
Dactyliophorae	Rhopilema	hispidum	20.84485363	106.6844037	Haiphong Bay, Vietnam	[94]
Dactyliophorae	Rhopilema	esculentum	5.075252162	99.65547819	Ipoh and Kuala Lumpur, Strait of Malacca	[94]
Dactyliophorae	Rhopilema	esculentum	2.698867684	111.4699954	Matu, Sarawak, Malaysia	[94]
Dactyliophorae	Rhopilema	sp.	4.231722995	100.5580664	Pangkor, Strait of Malacca	[94]
Dactyliophorae	Rhopilema	sp.	5.420030133	100.3250237	Penang, Strait of Malacca	[94]
Dactyliophorae	Rhopilema	hispidum	12.68644166	101.2808323	Rayong, Gulf of Thailand	[94]
Dactyliophorae	Rhopilema	hispidum	13.54400496	100.272174	Samut Sakhon, Gulf of Thailand	[94]
Dactyliophorae	Rhopilema	sp.	4.028576755	101.0160336	Telok Anson, Strait of Malacca	[94]
Dactyliophorae	Rhopilema	hispidum	19.75201833	107.7497424	Tongking Bay, Vietnam	[94]
Dactyliophorae	Rhopilema	hispidum	-6.895495909	112.0402914	Tuban, East Java, Java Sea, Indonesia	[94]
Dactyliophorae	Rhopilema	nomadica	36.29722811	30.14800422	Finike, SE Turkey	[152]
Dactyliophorae	Rhopilema	hispidum	3.16132216	101.2912872	Klang Strait, Selangor, Malayasia	[71]
Dactyliophorae	Rhopilema	esculentum	3.16132216	101.2912872	Klang Strait, Selangor, Malayasia	[71]
Dactyliophorae	Rhopilema	hispidum	4.183158304	100.5778917	Pulau Pangkor	[71]
Dactyliophorae	Rhopilema	hispidum	5.463828938	100.2076846	Teluk Bungah	[71]
Dactyliophorae	Rhopilema	esculentum	1.591447773	110.7042276	Sarawak	[153]
Dactyliophorae	Rhopilema	nomadica	36.64323895	22.83547938	Lakonikos Kolpos, Greece	[154]
Dactyliophorae	Rhopilema	nomadica	36.66878225	22.49961201	Skoutary, Greece	[154]
Dactyliophorae	Rhopilema	nomadica	32.82463944	35.02321284	Haifa Bay	[155]
Dactyliophorae	Rhopilema	esculentum	3.66166667	100.760222	Bagan Datoh, Perak, Malayasia	[112]
Dactyliophorae	Rhopilema	hispidum	3.66166667	100.760222	Bagan Datoh, Perak, Malayasia	[112]
Dactyliophorae	Rhopilema	hispidum	5.39272222	100.1619444	Balik Pulau, Penang, Malaysia	[112]
Dactyliophorae	Rhopilema	esculentum	5.39272222	100.1619444	Balik Pulau, Penang, Malaysia	[112]
Dactyliophorae	Rhopilema	hispidum	2.89736111	101.3054444	Carey Island, Selangor, Malayasia	[112]
Dactyliophorae	Rhopilema	esculentum	2.89736111	101.3054444	Carey Island, Selangor, Malayasia	[112]

Dactyliophorae	Rhopilema	hispidum	6.290472	99.725389	Cenang Beach, Langkawi, Kedah, Malaysia	[112]
Dactyliophorae	Rhopilema	hispidum	5.31488889	100.3051389	Jerejak Island, Penang, Malaysia	[112]
Dactyliophorae	Rhopilema	esculentum	5.31488889	100.3051389	Jerejak Island, Penang, Malaysia	[112]
Dactyliophorae	Rhopilema	hispidum	6.42163889	99.86694444	Kilim Geoforest Park, Langkawi, Kedah, Malaysia	[112]
Dactyliophorae	Rhopilema	hispidum	3.16855556	101.279	Klang Strait, Selangor, Malayasia	[112]
Dactyliophorae	Rhopilema	esculentum	3.16855556	101.279	Klang Strait, Selangor, Malayasia	[112]
Dactyliophorae	Rhopilema	hispidum	1.33458333	103.4370833	Kukup (southwest Johor)	[112]
Dactyliophorae	Rhopilema	hispidum	4.84063889	100.6217222	Matang Mangrove Reserve, Perak, Malayasia	[112]
Dactyliophorae	Rhopilema	esculentum	4.84063889	100.6217222	Matang Mangrove Reserve, Perak, Malayasia	[112]
Dactyliophorae	Rhopilema	hispidum	5.46975	100.2013333	Penang National Park, Penang, Malaysia	[112]
Dactyliophorae	Rhopilema	esculentum	5.46975	100.2013333	Penang National Park, Penang, Malaysia	[112]
Dactyliophorae	Rhopilema	hispidum	5.27305556	100.2858611	Teluk Tempoyak, Penang, Malaysia	[112]
Dactyliophorae	Rhopilema	esculentum	5.27305556	100.2858611	Teluk Tempoyak, Penang, Malaysia	[112]
Dactyliophorae	Rhopilema	esculentum	36.09027805	120.4081074	China	[156]
Dactyliophorae	Rhopilema	hispidum	36.09027805	120.4081074	China	[156]
Dactyliophorae	Rhopilema	hispidum	9.79566667	118.8281389	Indo-Pacific and Phillipines	[156]
Dactyliophorae	Rhopilema	esculentum	34.1321	133.2447	Inland Sea	[156]
Dactyliophorae	Rhopilema	asamushi	36.9276752	137.3515489	Japan Sea	[156]
Dactyliophorae	Rhopilema	asamushi	41.0784	141.0618	Mutsu Bay	[156]
Dactyliophorae	Rhopilema	esculentum	33.42639484	133.5626038	Pacific coasts of Honshu (Shikoku, Kyushu)	[156]
Dactyliophorae	Rhopilema	asamushi	33.42639484	133.5626038	Pacific coasts of Honshu (Shikoku, Kyushu)	[156]
Dactyliophorae	Rhopilema	hispidum	33.42639484	133.5626038	Pacific coasts of Honshu (Shikoku, Kyushu)	[156]
Dactyliophorae	Rhopilema	esculentum	37.76583015	126.314181	Ganghwado, Korea	[157]
Dactyliophorae	Rhopilema	hispidum	1.326960268	103.4467076	Kukup (southwest Johor)	[73]
Dactyliophorae	Rhopilema	esculentum	3.995	100.7602222	Bagan Datoh, Perak, Malayasia	[158]
Dactyliophorae	Rhopilema	hispidum	1.326960268	103.4467076	Kukup (southwest Johor)	[158]
Dactyliophorae	Rhopilema	esculentum	32.76550216	122.9042199	Coast of Ningbo, China	[159]
Dactyliophorae	Stomolophus	meleagris	27.85448021	-110.6325596	coastal lagoon Las Guasimas	[160]
Dactyliophorae	Stomolophus	meleagris	30.20344582	-87.80783936	Alabama, USA	[133]
Dactyliophorae	Stomolophus	meleagris	30.33599237	-81.39620703	Atlantic Florida, USA	[133]
Dactyliophorae	Stomolophus	meleagris	30.2	-88.19	Dauphin Island, AL, USA	[6]
Dactyliophorae	Stomolophus	meleagris	30.37	-81.4	Jacksonville Beach, FL, USA	[6]
Dactyliophorae	Stomolophus	meleagris	34.17341913	-89.60091375	Mississippi coast of the Gulf of Mexico	[10]
Dactyliophorae	Stomolophus	meleagris	33.54145394	-79.02890314	Murrells Inlet, South Carolina, USA	[161]
Dactyliophorae	Stomolophus	sp. 2	27.85091039	-110.5835479	Las Guásimas coastal lagoon	[162]
Dactyliophorae	Stomolophus	meleagris	18.41747	-93.45264	Arrastradero-Redondo, Mexico	[15]
Dactyliophorae	Stomolophus	sp. 2	24.17646333	-110.3157806	Bahía de la Paz, Mexico	[15]
Dactyliophorae	Stomolophus	sp. 2	27.86045002	-110.610733	Bahía Kino	[15]
Dactyliophorae	Stomolophus	sp. 6	27.86045002	-110.610733	Bahía Kino	[15]
Dactyliophorae	Stomolophus	sp. 2	29.79216741	-114.3783741	Bahía San Luis Gonzaga, Mexico	[15]
Dactyliophorae	Stomolophus	sp. 3	13.15890741	-88.0676713	Bocana del Esterón, El Savador	[15]
Dactyliophorae	Stomolophus	sp. 2	24.49134132	-110.2136243	Canal de San Lorenzo, Mexico	[15]
Dactyliophorae	Stomolophus	sp. 2	25.18893	-112.12913	Canal Principal, Mexico	[15]
Dactyliophorae	Stomolophus	meleagris	18.1747	-93.512	Carmen-Machona, Mexico	[15]
Dactyliophorae	Stomolophus	sp. 3	8.543578243	-79.87363432	Coronados, Panama	[15]
Dactyliophorae	Stomolophus	meleagris	30.23321389	-88.22995	Dauphin Island, USA	[15]
Dactyliophorae	Stomolophus	sp. 3	9.23620983	-83.86822983	Dominical, Coasta Rica	[15]
Dactyliophorae	Stomolophus	sp. 3	10.14959822	-85.13095944	Estero Culebras, Costa, Rica	[15]
Dactyliophorae	Stomolophus	sp. 5	14.02275972	-83.40319972	Estero Guililanding-Bilwi Tigni, Nicaragua	[15]
Dactyliophorae	Stomolophus	sp. 3	10.15251373	-85.10907421	Golfo de Nicoya, Costa Rica	[15]
Dactyliophorae	Stomolophus	sp. 1	31.66198596	-114.5757855	Golfo de Santa Clara, Mexico	[15]
Dactyliophorae	Stomolophus	sp. 5	14.08112	-83.39164983	Guililanding, Nicaragua	[15]
Dactyliophorae	Stomolophus	sp. 2	28.81058303	-111.9403109	Las Guásimas, Mexico	[15]
Dactyliophorae	Stomolophus	sp. 3	13.49486177	-89.8583513	Los Cóbanos, El Salvador	[15]
Dactyliophorae	Stomolophus	meleagris	18.21209722	-93.1012	Mecoacán, Mexico	[15]
Dactyliophorae	Stomolophus	sp. 2	26.8997269	-111.9605993	Mulegé	[15]
Dactyliophorae	Stomolophus	meleagris	35.03321389	-76.69954444	Oriental, USA	[15]
Dactyliophorae	Stomolophus	sp. 4	8.997337232	-79.48772808	Panamá Viejo, Panama	[15]
Dactyliophorae	Stomolophus	sp. 4	8.00048889	-79.49161111	Tocumen, Panama	[15]
Dactyliophorae	Stomolophus	sp. 5	14.10191972	-83.32129983	Tuapí, Nicaragua	[15]
Dactyliophorae	Stomolophus	meleagris	31.68691719	-114.4985037	Golfo de Santa Clara	[163]
Dactyliophorae	Stomolophus	meleagris	27.81799838	-110.4835727	Las Guásimas Lagoon, Sonora, México	[164]
Dactyliophorae	Stomolophus	meleagris	29.79456595	-84.31168785	Gulf of Mexico near Panacea,	[165]
Dactyliophorae	Stomolophus	meleagris	31.11307035	-81.40508224	Georgia coast, USA	[166]
Dactyliophorae	Stomolophus	meleagris	29.88979275	-84.49770301	North-eastern Gulf of Mexico, near FSU marine lab	[167]
Dactyliophorae	Stomolophus	meleagris	27.36184662	-110.465148	Eastern Gulf of California Mexico	[168]
Dactyliophorae	Stomolophus	meleagris	27.68023955	-110.5758233	Eastern Gulf of California Mexico	[168]
Dactyliophorae	Stomolophus	meleagris	-3.719107131	-38.50507132	Brazil, Ceará State, Fortaleza, Meireles beach	[104]
Dactyliophorae	Stomolophus	meleagris	27.18793778	-109.2678542	Las Guásimas, Sonora	[169]
Dactyliophorae	Stomolophus	meleagris	28.82182774	-111.9440972	Kino bay	[169]
Dactyliophorae	Stomolophus	meleagris	27.81799838	-110.4835727	Las Guásimas Lagoon, Sonora, México	[170]
Dactyliophorae	Stomolophus	meleagris	26.70515186	-109.5154938	Yávaros Bay	[170]
Dactyliophorae	Stomolophus	meleagris	27.83356099	-110.5000322	coastal plain of the state of Sonora, Mexico	[171]
Dactyliophorae	Stomolophus	meleagris	30.33141466	-88.49065672	Mississippi Sound	[172]
Dactyliophorae	Stomolophus	meleagris	9.333523856	-75.78337622	Cispatá Bay, off the region of San Antero, Córdoba	[173]
Dactyliophorae	Stomolophus	meleagris	24.20343903	-110.5292027	Bahía de La Paz, Baja California Sur, Mexico	[174]
Dactyliophorae	Stomolophus	meleagris	34.17997485	-77.80238882	Masonboro Inlet, North Carolina, USA	[175]
Dactyliophorae	Stomolophus	meleagris	28.31676398	-110.2983462	Las Guásimas Lagoon, Sonora, México	[176]
Dactyliophorae	Stomolophus	meleagris	-4.836659878	-37.24828742	Brazil, Rio Grande do Norte, Tibaú Beach	[111]
Dactyliophorae	Stomolophus	meleagris	24.84017524	66.90672034	Karachi West, Karachi City, Sindh, Pakistan	[177]
Dactyliophorae	Stomolophus	meleagris	30.33141466	-88.49065672	Mississippi Sound	[178]
Dactyliophorae	Stomolophus	meleagris	27.48770389	80.33144689	Fort Pierce Inlet, Indian river lagoon	[179]
Dactyliophorae	Stomolophus	meleagris	-25.49052949	-48.44524058	Baía de Paranaguá	[114]
Dactyliophorae	Stomolophus	meleagris	-25.01019926	-47.92801856	Cananéia	[114]
Dactyliophorae	Stomolophus	meleagris	-25.15521315	-47.91233703	Ilha do Camboriú, Brazil	[114]
Dactyliophorae	Stomolophus	meleagris	36.9609	10.4846	Tunis Gulf (SW Mediterranean)	[90]

Dactyliophorae	Stomolophus	meleagris	36.44477672	120.7988118	Aoshan Bay, Qingdao, Shandong Province, China	[180]

1. Ames, C.L.; Ohdera, A.H.; Colston, S.M.; Collins, A.G.; Fitt, W.K.; Morandini, A.C.; Erickson, J.S.; Vora, G.J. Fieldable Environmental DNA Sequencing to Assess Jellyfish Biodiversity in Nearshore Waters of the Florida Keys, United States. *Front. Mar. Sci.* **2021**, *8*, 640527, doi:10.3389/fmars.2021.640527.
2. Anthony, C.J.; Heagy, M.; Bentlage, B. Phenotypic Plasticity in Cassiopea Ornata (Cnidaria: Scyphozoa: Rhizostomeae) Suggests Environmentally Driven Morphology. *Zoomorphology* **2022**, *141*, 115–131, doi:10.1007/s00435-022-00558-4.
3. Arai, Y.; Gotoh, R.O.; Yokoyama, J.; Sato, C.; Okuizumi, K.; Hanzawa, N. Phylogenetic Relationships and Morphological Variations of Upside-down Jellyfishes, Cassiopea Spp. Inhabiting Palau Islands. *Biogeography* **2017**, *19*, 133–141.
4. Armani, A.; Tinacci, L.; Giusti, A.; Castigliego, L.; Gianfaldoni, D.; Guidi, A. What Is inside the Jar? Forensically Informative Nucleotide Sequencing (FINS) of a Short Mitochondrial COI Gene Fragment Reveals a High Percentage of Mislabeling in Jellyfish Food Products. *Food Res. Int.* **2013**, *54*, 1383–1393, doi:10.1016/j.foodres.2013.10.003.
5. Atta, M.M. The Scyphomedusae of the Mediterranean Coast of El Arish. *J. Egypt. Ger. Soc. Zool.* **1991**, *6*, 251–256.
6. Bayha, K.M.; Dawson, M.N.; Collins, A.G.; Barbeitos, M.S.; Haddock, S.H.D. Evolutionary Relationships Among Scyphozoan Jellyfish Families Based on Complete Taxon Sampling and Phylogenetic Analyses of 18S and 28S Ribosomal DNA. *Integr. Comp. Biol.* **2010**, *50*, 436–455, doi:10.1093/icb/icq074.
7. Bigelow, R.P. The Anatomy and Development of Cassiopea Xamachana. *Mem Boston Soc Nat Hist* **1900**, *5*, 191–193.
8. Browne, J.G. Parasites of Jellyfish in Eastern Australia. PhD Doctorate, Griffith University: Queensland, Australia, 2015.
9. Bruce, A.J. An Association between a Pontoniinid Shrimp and a Rhizostomatous Scyphozoan. *Crustaceana* **1972**, *23*, 300–302, doi:https://doi.org/10.1163/156854072X00192.
10. Burnett, J.W.; Calton, G.J.; Fenner, P.J.; Williamson, J.A. Serological Diagnosis of Jellyfish Envenomations. *Comp. Biochem. Physiol.* **1988**, *91C*, 79–83.
11. Carabantes, N.; Cerqueda-García, D.; García-Maldonado, J.Q.; Thomé, P.E. Changes in the Bacterial Community Associated With Experimental Symbiont Loss in the Mucus Layer of Cassiopea Xamachana Jellyfish. *Front. Mar. Sci.* **2022**, *9*, 879184, doi:10.3389/fmars.2022.879184.
12. Çevik, C.; Erkol, I.L.; Toklu, B. A New Record of an Alien Jellyfish from the Levantine Coast of Turkey - Cassiopea Andromeda (Forsskål, 1775) (Cnidaria: Scyphozoa:Rhizostomea). *Aquat. Invasions* **2006**, *1*, 196–197, doi:10.3391/ai.2006.1.3.18.
13. Chuang, S.H. *On Malayan Shores: A Log Cabin Book.*; Muwu Shosa: Singapore, 1961;
14. Cillari, T.; Allegra, A.; Berto, D.; Bosch-Belmar, M.; Falautano, M.; Maggio, T.; Milisenda, G.; Perzia, P.; Rampazzo, F.; Sinopoli, M.; et al. Snapshot of the

Distribution and Biology of Alien Jellyfish Cassiopea Andromeda (Forsskål, 1775) in a Mediterranean Touristic Harbour. *Biology* **2022**, *11*, 319, doi:10.3390/biology11020319.
15. Gómez Daglio, L.; Dawson, M.N. Species Richness of Jellyfishes (Scyphozoa : Discomedusae) in the Tropical Eastern Pacific: Missed Taxa, Molecules, and Morphology Match in a Biodiversity Hotspot. *Invertebr. Syst.* **2017**, *31*, 635, doi:10.1071/IS16055.
16. Dawson, M.N. Some Implications of Molecular Phylogenetics for Understanding Biodiversity in Jellyfishes, with Emphasis on Scyphozoa. *Hydrobiologia* **2004**, *530*, 249–260, doi:https://doi.org/10.1007/s10750-004-2659-3.
17. Deidun, A. Back with a Bang – an Unexpected Massive Bloom of Cassiopea Andromeda (Forskaal, 1775) in the Maltese Islands, Nine Years after Its First Appearance. *BioInvasions Rec.* **2018**, *7*, 399–404, doi:10.3391/bir.2018.7.4.07.
18. Durieux, D.M.; Du Clos, K.T.; Gemmell, B.J. Benthic Jellyfish Dominate Water Mixing in Mangrove Ecosystems. **2019**, *118*, e2025715118, doi:10.1073/pnas.2025715118.
19. Fitt, W.K.; Costley, K. The Role of Temperature in Survival of the Polyp Stage of the Tropical Rhizostome Jellyfish Cassiopea Xamachana. *J. Exp. Mar. Biol. Ecol.* **1998**, *222*, 79–91, doi:10.1016/S0022-0981(97)00139-1.
20. Gamero-Mora, E.; Halbauer, R.; Bartsch, V.; Stampar, S.N.; Morandini, A.C. Regenerative Capacity of the Upside-down Jellyfish Cassiopea Xamachana. *Zool. Stud.* **2019**, *58*, e37, doi:10.6620/ZS.2019.58-37.
21. Gamero-Mora, E.; Collins, A.G.; Boco, S.R.; Geson, S.M.; Morandini, A.C. Revealing Hidden Diversity among Upside-down Jellyfishes (Cnidaria: Scyphozoa: Rhizostomeae: *Invertebr. Syst.* **2022**, *36*, 63–89, doi:10.1071/IS21002.
22. Holland, B.S.; Dawson, M.N.; Crow, G.L.; Hofmann, D.K. Global Phylogeography of Cassiopea (Scyphozoa: Rhizostomeae): Molecular Evidence for Cryptic Species and Multiple Invasions of the Hawaiian Islands. *Mar. Biol.* **2004**, *145*, 1119–1128, doi:10.1007/s00227-004-1409-4.
23. Iliff, S.M.; Wilczek, E.R.; Harris, R.J.; Bouldin, R.; Stoner, E.W. Evidence of Microplastics from Benthic Jellyfish (Cassiopea Xamachana) in Florida Estuaries. *Mar. Pollut. Bull.* **2020**, *159*, 111521, doi:10.1016/j.marpolbul.2020.111521.
24. Karunarathne, K.D.; Liyanaarachchi, S.M.; De Croos, M.D.S.T. First Record of Upside-down Jellyfish Cassiopea Andromeda (Forskål, 1775) (Cnidaria: Scyphozoa: Rhizostomeae: Cassiopeidae) from Sri Lanka. *Sri Lanka J. Aquat. Sci.* **2020**, *25*, 57, doi:10.4038/sljas.v25i2.7577.
25. Kayal, E.; Bentlage, B.; Collins, A.G.; Kayal, M.; Pirro, S.; Lavrov, D.V. Evolution of Linear Mitochondrial Genomes in Medusozoan Cnidarians. *Genome Biol. Evol.* **2012**, *4*, 1–12, doi:10.1093/gbe/evr123.
26. Keable, S.J.; Ahyong, S.T. First Records of the Invasive "Upside-down Jellyfish", Cassiopea (Cnidaria: Scyphozoa: Rhizostomeae: Cassiopeidae), from Coastal Lakes of New South Wales, Australia. *Rec. Aust. Mus.* **2016**, *68*, 23–30, doi:10.3853/j.2201-4349.68.2016.1656.

27. Larson, R.J. Feeding Behaviour of Caribbean Scyphomedusae: Cassiopea Frondosa (Pallas) and Cassiopea Xamachana (Bigelow). *Stud. Nat Hist Caribb. Reg.* **1997**, *73*, 43–54.
28. Maggio, T.; Allegra, A.; Bosch-Belmar, M.; Cillari, T.; Cuttitta, A.; Falautano, M.; Milisenda, G.; Nicosia, A.; Perzia, P.; Sinopoli, M.; et al. Molecular Identity of the Non-Indigenous *Cassiopea* Sp. from Palermo Harbour (Central Mediterranean Sea). *J. Mar. Biol. Assoc. U. K.* **2019**, *99*, 1765–1773, doi:10.1017/S0025315419000924.
29. McKenzie, M.R.; Templeman, M.A.; Kingsford, M.J. Detecting Effects of Herbicide Runoff: The Use of Cassiopea Maremetens as a Biomonitor to Hexazinone. *Aquat. Toxicol.* **2020**, *221*, 105442, doi:10.1016/j.aquatox.2020.105442.
30. Nabipour, I.; Mohebbi, G.; Vatanpour, H.; Vazirizadeh, A. Hematological Parameters on the Effect of the Jellyfish Venom Cassiopea Andromeda in Animal Models. *Data Brief* **2017**, *11*, 517–521, doi:10.1016/j.dib.2017.02.054.
31. Newkirk, C.R.; Frazer, T.K.; Martindale, M.Q.; Schnitzler, C.E. Adaptation to Bleaching: Are Thermotolerant Symbiodiniaceae Strains More Successful Than Other Strains Under Elevated Temperatures in a Model Symbiotic Cnidarian? *Front. Microbiol.* **2020**, *11*, 822, doi:10.3389/fmicb.2020.00822.
32. Niggl, W.; Wild, C. Spatial Distribution of the Upside-down Jellyfish Cassiopea Sp. within Fringing Coral Reef Environments of the Northern Red Sea: Implications for Its Life Cycle. *Helgol. Mar. Res.* **2010**, *64*, 281–287, doi:10.1007/s10152-009-0181-8.
33. Niggl, W.; Naumann, M.S.; Struck, U.; Manasrah, R.; Wild, C. Organic Matter Release by the Benthic Upside-down Jellyfish Cassiopea Sp. Fuels Pelagic Food Webs in Coral Reefs. *J. Exp. Mar. Biol. Ecol.* **2010**, *384*, 99–106, doi:10.1016/j.jembe.2010.01.011.
34. Özgür, E. A Population of the Alien Jellyfish, Cassiopea Andromeda (Forsskål, 1775) [Cnidaria: Scyphozoa: Rhizostomea] in the Ölüdeniz Lagoon, Turkey. *Aquat. Invasions* **2008**, *3*, 423–428, doi:10.3391/ai.2008.3.4.8.
35. Prasade, A.; Nagale, P.; Apte, D. Cassiopea Andromeda (Forsskål, 1775) in the Gulf of Kutch, India: Initial Discovery of the Scyphistoma, and a Record of the Medusa in Nearly a Century. *Mar. Biodivers. Rec.* **2016**, *9*, 36, doi:10.1186/s41200-016-0031-8.
36. Radwan, F.F.Y.; Burnett, J.W.; Bloom, D.A.; Coliano, T.; Eldefrawi, M.E.; Erderly, H.; Aurelian, L.; Torres, M.; Heimer-de la Cotera, E.P. A Comparison of the Toxinological Characteristics of Two Cassiopea and Aurelia Species. *Toxicon* **2001**, *39*, 245–257, doi:10.1016/S0041-0101(00)00121-5.
37. De Rinaldis, G.; Leone, A.; De Domenico, S.; Bosch-Belmar, M.; Slizyte, R.; Milisenda, G.; Santucci, A.; Albano, C.; Piraino, S. Biochemical Characterization of Cassiopea Andromeda (Forsskål, 1775), Another Red Sea Jellyfish in the Western Mediterranean Sea. *Mar. Drugs* **2021**, *19*, 498, doi:10.3390/md19090498.
38. Schembri, P.J.; Deidun, A.; Vella, P.J. First Record of Cassiopea Andromeda (Scyphozoa: Rhizostomeae: Cassiopeidae) from the Central Mediterranean Sea. *Mar. Biodivers. Rec.* **2010**, *3*, e6, doi:10.1017/S1755267209990625.

39. Schiariti, A.; Morandini, A.; Jarms, G.; von Glehn Paes, R.; Franke, S.; Mianzan, H. Asexual Reproduction Strategies and Blooming Potential in Scyphozoa. *Mar. Ecol. Prog. Ser.* **2014**, *510*, 241–253, doi:10.3354/meps10798.
40. Stampar, S.N.; Gamero-Mora, E.; Maronna, M.M.; Fritscher, J.M.; Oliveira, B.S.P.; Sampaio, C.L.S.; Morandini, A.C. The Puzzling Occurrence of the Upside-down Jellyfish Cassiopea (Cnidaria: Scyphozoa) along the Brazilian Coast: A Result of Several Invasion Events? *Zoologia* **2020**, *37*, 1–10, doi:10.3897/zoologia.37.e50834.
41. Stoner, E.W.; Archer, S.K.; Layman, C.A. Increased Nutrient Availability Correlates with Increased Growth of the Benthic Jellyfish Cassiopea Spp. *Food Webs* **2022**, *31*, e00231, doi:10.1016/j.fooweb.2022.e00231.
42. Stoner, E.W.; Layman, C.A.; Yeager, L.A.; Hassett, H.M. Effects of Anthropogenic Disturbance on the Abundance and Size of Epibenthic Jellyfish Cassiopea Spp. *Mar. Pollut. Bull.* **2011**, *62*, 1109–1114, doi:10.1016/j.marpolbul.2011.03.023.
43. Stoner, E.W.; Yeager, L.A.; Sweatman, J.L.; Sebilian, S.S.; Layman, C.A. Modification of a Seagrass Community by Benthic Jellyfish Blooms and Nutrient Enrichment. *J. Exp. Mar. Biol. Ecol.* **2014**, *461*, 185–192, doi:10.1016/j.jembe.2014.08.005.
44. Stoner, E.W.; Sebilian, S.S.; Layman, C.A. Comparison of Zooxanthellae Densities from Upside-down Jellyfish, Cassiopea Xamachana, across Coastal Habitats of The Bahamas. *Rev. Biol. Mar. Oceanogr.* **2016**, *51*, 203–208, doi:10.4067/S0718-19572016000100022.
45. Uchida, T. Some Medusae from the Central Pacific (With 13 Textfigures). *J. Fac. Sci. Hokkaido Univ. Ser. VI* **1947**, *9*, 297–319.
46. Wood, E.M.; Aw, M. *Reef Fishes, Corals and Invertebrates of the South China Sea: Including Thailand, Hong Kong, China, Malaysia, Taiwan, Singapore, Indonesia and the Philippines.*; New Holland Publishers: New Holland, London, 2002;
47. Zarnoch, C.B.; Hossain, N.; Fusco, E.; Alldred, M.; Hoellein, T.J.; Perdikaris, S. Size and Density of Upside-down Jellyfish, Cassiopea Sp., and Their Impact on Benthic Fluxes in a Caribbean Lagoon. *Mar. Environ. Res.* **2020**, *154*, 104845, doi:10.1016/j.marenvres.2019.104845.
48. Behera, D.P.; Thirumaran, G.; Sahu, B.K.; Das, R.R. High Occurrence of Jellyfish (Catostylus Perezi, Ranson 1945) in Proximity to Industrial Development, Gulf of Kutch/ Kachchh, India. *Indian J. Geo-Mar. Sci.* **2022**, *51*, doi:10.56042/ijms.v51i06.41458.
49. Boco, S.R.; Metillo, E.B.; Papa, R.D.S. Abundance, Size and Symbionts of Catostylus Sp. Medusae (Scyphozoa, Rhizostomeae) in Panguil Bay, Northern Mindanao, Philippines. *Philipp. J. Syst. Biol.* **2014**, *8*.
50. Browne, J.G.; Kingsford, M.J. A Commensal Relationship between the Scyphozoan Medusae Catostylus Mosaicus and the Copepod Paramacrochiron Maximum. *Mar. Biol.* **2005**, *146*, 1157–1168, doi:10.1007/s00227-004-1517-1.
51. Du, C.; He, J.; Sun, T.; Wang, L.; Wang, F.; Dong, Z. Molecular Identification on the Causative Species Jellyfish Blooms in the Northern South China Sea in 2019. *J. Trop. Oceanogr.* **2022**, *41*, 142–148, doi:10.11978/2021071.

52. Cruz, J.; Cerveira, I.; Andrade, I.; Baptista, V.; TeodÓsio, M.A. Prey Selectivity and Feeding Rates of the Scyphozoan *Catostylus Tagi* (Haeckel, 1869). *J. Plankton Res.* **2021**, *43*, 986–990, doi:10.1093/plankt/fbab065.
53. Gómez Daglio, L.; Hayati, R.; Coleman, T.; Han, Y.-M.; Muzaki, F.; Aunurohim; de Bellard, M.E.; Saptarini, D. Species Composition of Discomedusae Jellyfish (Scyphozoa) in the Coastal Waters of Eastern Surabaya, East Java. *Mar. Biodivers.* **2022**, *52*, 23, doi:10.1007/s12526-021-01253-1.
54. Dawson, M.N. Incipient Speciation of Catostylus Mosaicus (Scyphozoa, Rhizostomeae, Catostylidae), Comparative Phylogeography and Biogeography in South-East Australia. *J. Biogeogr.* **2005**, *32*, 515–533, doi:10.1111/j.1365-2699.2004.01193.x.
55. Govindan, S.; Ramanibai, R. Impacts of Climate Change on Hydropower Potential in Kaligandaki River Basin -A Case Study of Kaligandaki Gorge Hydropower Project, North of Nepal. *Oceanogr. Fish. Open Access J.* **2017**, *4*, doi:10.19080/OFOAJ.2017.04.555632.
56. Gul, S.; Morandini, A.C. New Records of Scyphomedusae from Pakistan Coast: Catostylus Perezi and Pelagia Cf. Noctiluca (Cnidaria: Scyphozoa). *Mar. Biodivers. Rec.* **2013**, *6*, e86, doi:10.1017/S1755267213000602.
57. Gul, S.; Jahangir, S.; Schiariti, A. Jellyfish Fishery in Pakistan. *Plankton Benthos Res.* **2015**, *10*, 220–224, doi:10.3800/pbr.10.220.
58. Hudson, J.R.; Bridge, N.F.; Walker, T.I. *Feasibility Study for Establishment of a Victorian Commercial Jellyfish Fishery*; FRDC Project ; 92/125.31.; Marine and Freshwater Resources Institute: Queenscliff, Vic., 1997; ISBN 978-0-7306-6294-5.
59. Klun, K.; Slizyte, R. *Chemical Composition of JF Mucus*; GoJelly EU Project; 2021; p. 35;.
60. Kwang, S.Y.; Yahya, K.; Talib, A. The Monthly Distribution and Abundance of Jellyfish (Medusa) Species in the Coastal Waters of Penang National Park, Penang, Malaysia.; Universiti Kebangsaan Malaysia and National Oceanographic Directorate: Bangi, Malaysia; pp. 228–239.
61. Loveridge, A.; Pitt, K.A.; Lucas, C.H.; Warnken, J. Extreme Changes in Salinity Drive Population Dynamics of Catostylus Mosaicus Medusae in a Modified Estuary. *Mar Env. Res* **2021**, *168*, 105306, doi:10.1016/j.marenvres.2021.105306.
62. Mayer, A.G. Medusae of the Philippines and of Torres Strait. *Pap. Tortugas Lab. Carnegie Inst. Wash.* **1915**, *8212*, 157–202.
63. Moestafa, S.H.; McConnaughey, B. Catostylus Ouwensi (Rhizostomeae, Catostylidae), a New Jellyfish from Irian (New Guinea) and Ouwensia Catostyli n, Gen., n, Sp., Parasitic in C.Ouwensi. *Treubia* **1966**, *27*, doi:10.14203/treubia.v27i1.1551.
64. Morais, Z.B.; Pintão, A.M.; Costa, I.M.; Calejo, M.T.; Bandarra, N.M.; Abreu, P. Composition and In Vitro Antioxidant Effects of Jellyfish *Catostylus Tagi* from Sado Estuary (SW Portugal). *J. Aquat. Food Prod. Technol.* **2009**, *18*, 90–107, doi:10.1080/10498850802581799.

65. Ng, P.K.L.; Corlett, R.T. *Singapore Biodiversity: An Encyclopedia of the Natural Environment and Sustainable Development.*; Tan, H.T.W., Ed.; Editions Didier Millet and Raffles Museum of Biodiversity Research: National University of Singapore, Singapore, 2011;
66. Parracho, T.; Morais, Z. Catostylus Tagi: Partial RDNA Sequencing and Characterisation of Nematocyte Structures Using Two Improvements in Jellyfish Sample Preparation. *J. Venom. Anim. Toxins Trop. Dis.* **2015**, *21*, 40, doi:10.1186/s40409-015-0037-4.
67. Peach, M.B.; Pitt, K.A. Morphology of the Nematocysts of the Medusae of Two Scyphozoans, Catostylus Mosaicus and Phyllorhiza Punctata (Rhizostomeae): Implications for Capture of Prey: Nematocysts and Prey of Rhizostome Jellyfish. *Invertebr. Biol.* **2005**, *124*, 98–108, doi:10.1111/j.1744-7410.2005.00012.x.
68. Pitt, K.A.; Kingsford, M.J. Reproductive Biology of the Edible Jelly®sh Catostylus Mosaicus (Rhizostomeae). *Mar. Biol.* **2000**, *137*, 791–799, doi:10.1007/s002270000399.
69. Pitt, K.A.; Koop, K.; Rissik, D. Contrasting Contributions to Inorganic Nutrient Recycling by the Co-Occurring Jellyfishes, Catostylus Mosaicus and Phyllorhiza Punctata (Scyphozoa, Rhizostomeae). *J. Exp. Mar. Biol. Ecol.* **2005**, *315*, 71–86, doi:10.1016/j.jembe.2004.09.007.
70. Riyas, A.; Kumar, A.B.; Vakani, B. First Record of Rhizostome Jellyfish Catostylus Perezi Ranson 1945 (Cnidaria: Scyphozoa) from the Indian Coast. *Thalass. Int. J. Mar. Sci.* **2019**, *35*, 519–524, doi:10.1007/s41208-019-00157-z.
71. Rizman-Idid, M.; Farrah-Azwa, A.B.; Chong, V.C. Preliminary Taxonomic Survey and Molecular Documentation of Jellyfish Species (Cnidaria: Scyphozoa and Cubozoa) in Malaysia. *Zool. Stud.* **2016**, *55*, doi:10.6620/ZS.2016.55-35.
72. Rodrigues, T.F.C. Jellyfish Impact on Aquatic Ecosystems: Warning for the Development of Mass Occurrences Early Detection Tools. Master in Biology and Management of Water Quality, Universidade do Porto: Porto, Portugal, 2019.
73. Williamson, J.A.; Burnett, J.W.; Fenner, P.J.; Rifkin, J.F. *Venomous and Poisonous Marine Animals: A Medical and Biological Handbook.*; Surf life Saving Queensland Inc. University of New South Wales Press Ltd: Kensington, Australia, Brisbane: Queensland, 1996;
74. Astorga, D.; Ruiz, J.; Prieto, L. Ecological Aspects of Early Life Stages of Cotylorhiza Tuberculata (Scyphozoa: Rhizostomae) Affecting Its Pelagic Population Success. *Hydrobiologia* **2012**, *690*, 141–155, doi:10.1007/s10750-012-1036-x.
75. Balık, S. *İzmir Körfezi Ye Civarında Bulunan Deniz Analarının (Scyphozoa, Coelenterata) Taksonomik Ye Ekolojik Özellikleri Üzerine Araştırmalar*; Ege Üniversitesi Fen Fakültesi ilmi Raporlar Serisi; Ege Üniversitesi: Bornova, İzmir, Türkiye, 1973; pp. 1–15;.
76. Çevik, C.; Derici, O.; Cevik, F.; Cavas, L. First Record of Phyllorhiza Punctata von Lendenfeld, 1884 (Scyphozoa: Rhizostomeae: Mastigiidae) from Turkey. *Aquat. Invasions* **2011**, *6*, S27–S28, doi:10.3391/ai.2011.6.S1.006.

77. Cortés-Lara, S.; Urdiain, M.; Mora-Ruiz, M.; Prieto, L.; Rosselló-Móra, R. Prokaryotic Microbiota in the Digestive Cavity of the Jellyfish Cotylorhiza Tuberculata. *Syst. Appl. Microbiol.* **2015**, *38*, 494–500, doi:10.1016/j.syapm.2015.07.001.
78. Dall'Olio, L.R.; Beran, A.; Flander-Putrle, V.; Malej, A.; Ramšak, A. Diversity of Dinoflagellate Symbionts in Scyphozoan Hosts From Shallow Environments: The Mediterranean Sea and Cabo Frio (Rio de Janeiro, Brazil). *Front. Mar. Sci.* **2022**, *9*, 867554, doi:10.3389/fmars.2022.867554.
79. Enrique-Navarro, A.; Huertas, E.; Flander-Putrle, V.; Bartual, A.; Navarro, G.; Ruiz, J.; Malej, A.; Prieto, L. Living Inside a Jellyfish: The Symbiosis Case Study of Host-Specialized Dinoflagellates, "Zooxanthellae", and the Scyphozoan Cotylorhiza Tuberculata. *Front. Mar. Sci.* **2022**, *9*, 817312, doi:10.3389/fmars.2022.817312.
80. Fernández-Alías, A.; Marcos, C.; Quispe, J.I.; Sabah, S.; Pérez-Ruzafa, A. Population Dynamics and Growth in Three Scyphozoan Jellyfishes, and Their Relationship with Environmental Conditions in a Coastal Lagoon. *Estuar. Coast. Shelf Sci.* **2020**, *243*, 106901, doi:10.1016/j.ecss.2020.106901.
81. Galil, B.S.; Gershwin, L.-A.; Zorea, M.; Rahav, A.; Rothman, S.B.-S.; Fine, M.; Lubinevsky, H.; Douek, J.; Paz, G.; Rinkevich, B. Cotylorhiza Erythraea Stiasny, 1920 (Scyphozoa: Rhizostomeae: Cepheidae), yet Another Erythraean Jellyfish from the Mediterranean Coast of Israel. *Mar. Biodivers.* **2017**, *47*, 229–235, doi:10.1007/s12526-016-0449-6.
82. İşinibilir, M. First Record of Cotylorhiza Tuberculata (Macri, 1778) from the Sea of Marmara. *Aquat. Sci. Eng.* **2020**, *36*, 38–41, doi:10.26650/ASE2020804717.
83. Kikinger, R. Cotylorhiza Tuberculata (Cnidaria: Scyphozoa) - Life History of a Stationary Population. *Mar. Ecol.* **1992**, *13*, 333–362, doi:10.1111/j.1439-0485.1992.tb00359.x.
84. LaJeunesse, T.C.; Casado-Amezúa, P.; Hume, B.C.C.; Butler, C.C.; Mordret, S.; Piredda, R.; De Luca, P.; Pannone, R.; Sarno, D.; Wiedenmann, J.; et al. Mutualistic Dinoflagellates with Big Disparities in Ribosomal DNA Variation May Confound Estimates of Symbiont Diversity and Ecology in the Jellyfish Cotylorhiza Tuberculata. *Symbiosis* **2022**, *88*, 1–10, doi:10.1007/s13199-022-00880-x.
85. Lengar, Ž.; Klun, K.; Dogsa, I.; Rotter, A.; Stopar, D. Sequestration of Polystyrene Microplastics by Jellyfish Mucus. *Front. Mar. Sci.* **2021**, *8*, 690749, doi:10.3389/fmars.2021.690749.
86. Leone, A.; Lecci, R.; Durante, M.; Piraino, S. Extract from the Zooxanthellate Jellyfish Cotylorhiza Tuberculata Modulates Gap Junction Intercellular Communication in Human Cell Cultures. *Mar. Drugs* **2013**, *11*, 1728–1762, doi:10.3390/md11051728.
87. Leone, A.; Lecci, R.; Durante, M.; Meli, F.; Piraino, S. The Bright Side of Gelatinous Blooms: Nutraceutical Value and Antioxidant Properties of Three

Mediterranean Jellyfish (Scyphozoa). *Mar. Drugs* **2015**, *13*, 4654–4681, doi:10.3390/md13084654.
88. Leone, A.; Lecci, R.M.; Milisenda, G.; Piraino, S. Mediterranean Jellyfish as Novel Food: Effects of Thermal Processing on Antioxidant, Phenolic, and Protein Contents. *Eur. Food Res. Technol.* **2019**, *245*, 1611–1627, doi:10.1007/s00217-019-03248-6.
89. Mir-Arguimbau, J.; Sabatés, A.; Tilves, U. Trophic Ecology of Trachurus Mediterraneus Juveniles Associated with the Jellyfish Rhizostoma Pulmo and Cotylorhiza Tuberculata. *J. Sea Res.* **2019**, *147*, 28–36, doi:10.1016/j.seares.2019.02.004.
90. Yahia, M.N.D.; Goy, J.; Yahia-Kéfi, O.D. Distribution and ecology of Medusae and Scyphomedusae (Cnidaria) in Tunis Gulf (SW Mediterranean). *Oceanol. Acta* **2003**, *26*, 645–655, doi:10.1016/j.oceact.2003.05.002.
91. Hubot, N.; Giering, S.L.C.; Lucas, C.H. Similarities between the Biochemical Composition of Jellyfish Body and Mucus. *J. Plankton Res.* **2022**, *44*, 337–344, doi:10.1093/plankt/fbab091.
92. Jordaan, R.K.; Somers, M.J.; Hall, G.; McIntyre, T. Plasticity and Specialisation in the Isotopic Niche of African Clawless Otters Foraging in Marine and Freshwater Habitats. *Mamm. Biol.* **2019**, *98*, 61–72, doi:10.1016/j.mambio.2019.07.006.
93. Pages, F.; Gili, J.-M.; Bouillon, J. Medusae (Hydrozoa, Scyphozoa, Cubozoa) of the Benguela Current (Southeastern Atlantic). *Sci. Mar.* **1992**, *56*, 1–64.
94. Omori, M.; Nakano, E. Jellyfish Fisheries in Southeast Asia. In *Jellyfish Blooms: Ecological and Societal Importance*; Purcell, J.E., Graham, W.M., Dumont, H.J., Eds.; Springer Netherlands: Dordrecht, 2001; pp. 19–26 ISBN 978-94-010-3835-5.
95. Light, S.F. Some Philippine Scyphomedusae, Including Two New Genera, Five New Species, and One New Variety. *Philipp. J. Sci.* **1914**, *9*, 195–231.
96. Vichaya, K. *A survey and identification of jellyfish in coastal area, Chonburi province*; Income budget research project (government subsidies) Fiscal Year 2019; Burapha University, 2020;
97. Banha, T.N.S.; Morandini, A.C.; Rosário, R.P.; Martinelli Filho, J.E. Scyphozoan Jellyfish (Cnidaria, Medusozoa) from Amazon Coast: Distribution, Temporal Variation and Length–Weight Relationship. *J. Plankton Res.* **2020**, *42*, 767–778, doi:10.1093/plankt/fbaa056.
98. Cedeño Posso, C.M.C.-P.; Lecompte Pérez, O.P. PRIMER REGISTRO DE MEDUSAS DEL GÉNERO LYCHNORHIZA (CNIDARIA: SCYPHOZOA: RHIZOSTOMEAE: LYCHNORHIZIDAE) EN EL MAR CARIBE COLOMBIANO. *Bull. Mar. Coast. Res.* **2016**, *42*, doi:10.25268/bimc.invemar.2013.42.2.42.
99. Júnior, M.N.; Haddad, M.A. Lychnorhiza Lucerna Haeckel (Scyphozoa, Rhizostomeae) and Libinia Ferreirae Brito Capello (Decapoda, Majidae) Association in Southern Brazil. *Rev. Bras. Zool.* **2005**, *22*, 908–912, doi:10.1590/S0101-81752005000400015.

100. Junior, M.N.; Silva, J.D.L.E. Associações entre medusas (Cnidaria) e isópodos (Crustacea) nos litorais do Paraná e Santa Catarina, Brasil. *Acta Biológica Parana.* **2005**, *34*, 127–138, doi:10.5380/abpr.v34i0.959.
101. Júnior, M.N.; Nagata, R.M.; Haddad, M.A. Seasonal Variation of Macromedusae (Cnidaria) at North Bay, Florianópolis, Southern Brazil. *Zool. Curitiba* **2010**, *27*, 377–386, doi:10.1590/S1984-46702010000300009.
102. Liu, R.; Xiao, J.; Zhang, X.; Charatsee, A. Genetic analysis of common venomous Cubozoa and Scyphozoa in Thailand waters [J]. *Haiyang Xuebao* **2016**, *38*, 51–61, doi:10.3969/j.issn.0253-4193.2016.06.006.
103. Morandini, A.C.; Martorelli, S.R.; Marques, A.C.; Silveira, F.L. da Digenean Metacercaria (Trematoda, Digenea, Lepocreadiidae) Parasitizing "Coelenterates" (Cnidaria, Scyphozoa and Ctenophora) from Southeastern Brazil. *Braz. J. Oceanogr.* **2005**, *53*, 39–45, doi:10.1590/S1679-87592005000100004.
104. Morandini, A.C.; Soares, M. de O.; Matthews-Cascon, H.; Marques, A.C. A Survey of the Scyphozoa and Cubozoa (Cnidaria, Medusozoa) from the Ceará Coast (NE Brazil). *Biota Neotropica* **2006**, *6*, doi:10.1590/S1676-06032006000200021.
105. Nagata, R.M.; Morandini, A.C. Diet, Prey Selection, and Individual Feeding Rates of the Jellyfish Lychnorhiza Lucerna (Scyphozoa, Rhizostomeae). *Mar. Biol.* **2018**, *165*, 187, doi:10.1007/s00227-018-3445-5.
106. Nagata, R.; Morandini, A.; Colin, S.; Migotto, A.; Costello, J. Transitions in Morphologies, Fluid Regimes, and Feeding Mechanisms during Development of the Medusa Lychnorhiza Lucerna. *Mar. Ecol. Prog. Ser.* **2016**, *557*, 145–159, doi:10.3354/meps11855.
107. Riyas, A.; Kumar, A.; Chandran, M.; Jaleel, A.; Biju Kumar, A. The Venom Proteome of Three Common Scyphozoan Jellyfishes (Chrysaora Caliparea, Cyanea Nozakii and Lychnorhiza Malayensis) (Cnidaria: Scyphozoa) from the Coastal Waters of India. *Toxicon* **2021**, *195*, 93–103, doi:10.1016/j.toxicon.2021.03.005.
108. Santos, L.D.A.; Feres, S.J.C.; Lopes, A.T.L. PRIMEIRO REGISTRO DA ASSOCIAÇÃO ENTRE O CARANGUEJO Libinia Ferreirae (CRUSTACEA; DECAPODA; MAJIDAE) E A Medusa Lychnorhiza Lucerna (CNIDARIA; SCYPHOZOA; RHIZOSTOMEAE) PARA O GOLFÃO MARANHENSE. *Bol Lab Hidrobiol* **2014**, *21*.
109. Schiariti, A.; Kawahara, M.; Uye, S.; Mianzan, H.W. Life Cycle of the Jellyfish Lychnorhiza Lucerna (Scyphozoa: Rhizostomeae). *Mar. Biol.* **2008**, *156*, 1–12, doi:10.1007/s00227-008-1050-8.
110. Schiariti, A.; Christiansen, E.; Morandini, A.C.; da Silveira, F.L.; Giberto, D.A.; Mianzan, H.W. Reproductive Biology of *Lychnorhiza Lucerna* (Cnidaria: Scyphozoa: Rhizostomeae): Individual Traits Related to Sexual Reproduction. *Mar. Biol. Res.* **2012**, *8*, 255–264, doi:10.1080/17451000.2011.616897.
111. Soares, M. de O. Ecology of Jellyfishes (Cnidaria: Scyphozoa, Cubozoa) in Mucurip Cove, Fortaleza, Northeast of Brazil. Master's degree in Tropical Marine Sciences, Universidade Federal do Ceará: Fortaleza, Brazil, 2007.

112. Syazwan, W.M.; Rizman-Idid, M.; Low, L.B.; Then, A.Y.-H.; Chong, V.C. Assessment of Scyphozoan Diversity, Distribution and Blooms: Implications of Jellyfish Outbreaks to the Environment and Human Welfare in Malaysia. *Reg. Stud. Mar. Sci.* **2020**, *39*, 101444, doi:10.1016/j.rsma.2020.101444.
113. Syazwan, W.M.; Low, L.B.; Rizman-Idid, M. First Record in Peninsular Malaysia and Morphological Redescription of Lychnorhiza Malayensis (Scyphozoa: Rhizostomeae: Lychnorhizidae). *Raffles Bull. Zool.* **2020**, *68*, 3249, doi:10.26107/RBZ-2020-0004.
114. Vannucci, M. Hydrozoa e Scyphozoa Existentes No Instituto Oceanográfico II. *Bol. Inst. Oceanogr.* **1954**, *5*, doi:https://doi.org/10.1590/S0373-55241954000100005.
115. Zamponi, M.O. The Association between Medusa Lychnorhiza Lucerna (Scyphomedusae, Rhizostomeae) and Decapod Libinia Spinosa (Brachyura, Majidae) Recorded for the First Time in Neritic Waters of Argentina. *Russ. J. Mar. Biol. Vol.* **2002**, *28*, 267–268.
116. Browne, J.G.; Pitt, K.A.; Cribb, T.H. DNA Sequencing Demonstrates the Importance of Jellyfish in Life Cycles of Lepocreadiid Trematodes. *J. Helminthol.* **2020**, *94*, e182, doi:10.1017/S0022149X20000632.
117. Fancett, M.S.; Jenkins, G.P. Predatory Impact of Scyphomedusae on Ichthyoplankton and Other Zooplankton in Port Phillip Bay. *J. Exp. Mar. Biol. Ecol.* **1988**, *116*, 63–77, doi:10.1016/0022-0981(88)90246-8.
118. Basso, L.; Rizzo, L.; Piraino, S.; Stabili, L. Metabolic Diversity of Microbial Community Associated with Rhizostoma Pulmo (Scyphozoa: Rhizostomeae). *J. Mar. Microbiol.* **2017**, *1*, 5–8.
119. Ben Faleh, A.R.; Allaya, H.; Armani, A.; Shahin, A.A.B. Significant Genetic Differentiation among Meroplanktonic Barrel Jellyfish *Rhizostoma Pulmo* (Cnidaria: Scyphozoa) in the Mediterranean Sea. *Afr. J. Mar. Sci.* **2017**, *39*, 1–8, doi:10.2989/1814232X.2017.1303395.
120. Fuentes, V.; Straehler-Pohl, I.; Atienza, D.; Franco, I.; Tilves, U.; Gentile, M.; Acevedo, M.; Olariaga, A.; Gili, J.-M. Life Cycle of the Jellyfish Rhizostoma Pulmo (Scyphozoa: Rhizostomeae) and Its Distribution, Seasonality and Inter-Annual Variability along the Catalan Coast and the Mar Menor (Spain, NW Mediterranean). *Mar. Biol.* **2011**, *158*, 2247–2266, doi:10.1007/s00227-011-1730-7.
121. Holst, S.; Sötje, I.; Tiemann, H.; Jarms, G. Life Cycle of the Rhizostome Jellyfish Rhizostoma Octopus (L.) (Scyphozoa, Rhizostomeae), with Studies on Cnidocysts and Statoliths. *Mar. Biol.* **2007**, *151*, 1695–1710, doi:10.1007/s00227-006-0594-8.
122. Holst, S. Grundlagen der Populationsentwicklung verschiedener Scyphozoa (Cnidaria) der Deutschen Bucht. PhD Doctorate, Universität Hamburg: Hamburg, Germany, 2008.
123. Kienberger, K.; Riera-Buch, M.; Schönemann, A.M.; Bartsch, V.; Halbauer, R.; Prieto, L. First Description of the Life Cycle of the Jellyfish Rhizostoma Luteum (Scyphozoa: Rhizostomeae). *PLOS ONE* **2018**, *13*, e0202093, doi:10.1371/journal.pone.0202093.

124. Lee, P.L.M.; Dawson, M.N.; Neill, S.P.; Robins, P.E.; Houghton, J.D.R.; Doyle, T.K.; Hays, G.C. Identification of Genetically and Oceanographically Distinct Blooms of Jellyfish. *J. R. Soc. Interface* **2013**, *10*, 20120920, doi:10.1098/rsif.2012.0920.
125. Lilley, M.K.S.; Houghton, J.D.R.; Hays, G.C. Distribution, Extent of Inter-Annual Variability and Diet of the Bloom-Forming Jellyfish *Rhizostoma* in European Waters. *J. Mar. Biol. Assoc. U. K.* **2009**, *89*, 39–48, doi:10.1017/S0025315408002439.
126. Mirzoyan, Z.A.; Martynyuk, M.L.; Khrenkin, D.V.; Afanasyev, D.F. DEVELOPMENT OF THE SCYPHOZOAN JELLYFISH *RHIZOSTOMA PULMO* AND *AURELIA AURITA* POPULATIONS IN THE AZOV SEA. *Водные Биоресурсы И Среда Обитания* **2019**, *2*, 27–35, doi:10.47921/2619-1024_2019_2_2_27.
127. Muhammed, F.; Sultana, R. New Record of Edible Jellyfish, Rhizostoma Pulmo (Cnidaria: Scyphozoa: Rhizostomitidae) from Pakistani Waters. *Mar. Biodivers. Rec.* **2008**, *1*, e67, doi:10.1017/S1755267207007294.
128. O'Connor, B.; McGrath, D. On the Occurrence of the Scyphozoan Rhizostoma Octopus (L.) around the Irish Coast in 1976. *Ir. Nat. J.* **1978**, *19*, 261–263.
129. Peric, M. Comparison of Mitochondrial and Nuclear Genetic Markers of Barrell Jellyfish (Rhizostoma Pulmo) From North Adriatic and Central Mediterrean Biogeographic Region. Master, University of Primorska, 2012.
130. Ramšak, A.; Stopar, K.; Malej, A. Comparative Phylogeography of Meroplanktonic Species, Aurelia Spp. and Rhizostoma Pulmo (Cnidaria: Scyphozoa) in European Seas. *Hydrobiologia* **2012**, *690*, 69–80, doi:10.1007/s10750-012-1053-9.
131. Avsar, D.; Çevik, C.; Türeli, C. İskenderun Körfezi Için Yeni Bir Tür Olan (Rhopilema Nomadica)'nın Biyometrisi ve Yumurtalık Koyundaki Bulunurluğu. *Ulus. Biyol. Kongresi* **1996**, *13*, 17–20.
132. Awong, H.; Ibrahim, S.; Ambak, M.A.; Samo, K. Jellyfish (Semi-China Type) Processing for Human Consumptions in Darvel Bay, Sabah, Malaysia. *Borneo Res J* **2010**, *4*, 59–70.
133. Bayha, K.M.; Graham, W.M. A New Taqman PCR-Based Method for the Detection and Identification of Scyphozoan Jellyfish Polyps. *Hydrobiologia* **2009**, *616*, 217–228, doi:10.1007/s10750-008-9590-y.
134. Calder, D.R. Nematocysts of the Medusa Stage of Rhopilema Verrilli (Scyphozoa, Rhizostomeae). *Trans. Am. Microsc. Soc.* **1972**, *91*, 213, doi:10.2307/3225411.
135. Deidun, A.; Arrigo, S.; Piraino, S. The Westernmost Record of Rhopilema Nomadica (Galil, 1990) in the Mediterranean – off the Maltese Islands. *Aquat. Invasions* **2011**, *6*, S99–S103, doi:10.3391/ai.2011.6.S1.023.
136. Feng, J.; Yu, H.; Li, C.; Xing, R.; Liu, S.; Wang, L.; Cai, S.; Li, P. Isolation and Characterization of Venom from Nematocysts of Jellyfish Rhopilema Esculentum

Kishinouye. *Chin. J. Oceanol. Limnol.* **2009**, *27*, 869–874, doi:10.1007/s00343-009-9225-9.
137. Galil, B.; Zenetos, A. A Sea Change – Exotics in the Eastern Mediterranean Sea. In: Leppakoski E, Gollasch S, Olenin S (Eds) Invasive Aquatic Species in Europe. In *Distribution, Impacts and management*; Kluwer Academic Publishers: Dordrecht, Boston, London, 2002; pp. 325–336.
138. Galil, B.; Spanier, E.; Ferguson, W. The Scyphomedusae of the Israeli Mediterranean Coast, Including Two Lessepsian Migrants to the Mediterranean. *Zool. Mededlingen* **1990**, *64*, 95–105.
139. Gülşahin, N.; Tarkan, A.N. The First Confirmed Record of the Alien Jellyfish Rhopilema Nomadica Galil, 1990 from the Southern Aegean Coast of Turkey. *Aquat. Invasions* **2011**, *6*, 95–97, doi:http://dx.doi.org/10.3391/ai.2011.6.S1.022.
140. Iida, A.; Nohara, K.; Yusoff, F.Md.; Srinui, K.; Ha, T.M.; Ohtsuka, S.; Metillo, E.B.; Pagliawan, H.B.; Nishikawa, J. Genetic Diversities of Commercially Harvested Jellyfish, *Rhopilema Hispidum* and *Lobonemoides Robustus* in Southeast Asia. *Plankton Benthos Res.* **2021**, *16*, 308–317, doi:10.3800/pbr.16.308.
141. Khong, N.M.H.; Yusoff, F.Md.; Jamilah, B.; Basri, M.; Maznah, I.; Chan, K.W.; Nishikawa, J. Nutritional Composition and Total Collagen Content of Three Commercially Important Edible Jellyfish. *Food Chem.* **2016**, *196*, 953–960, doi:10.1016/j.foodchem.2015.09.094.
142. Kideys, A.E.; Gucu, A.C. Rhopilema Nomadica: A Lessepsian Scyphomedusan New to the Mediterranean Coast of Turkey. *Isr. J. Zool.* **1995**, *41*, 615–617.
143. Kitamura, M.; Omori, M. Synopsis of Edible Jellyfishes Collected from Southeast Asia, with Notes on Jellyfish Fisheries. *Plankton Benthos Res.* **2010**, *5*, 106–118, doi:10.3800/pbr.5.106.
144. Kuplik, Z.; Angel, D.L. Diet Composition and Some Observations on the Feeding Ecology of the Rhizostome *Rhopilema Nomadica* in Israeli Coastal Waters. *J. Mar. Biol. Assoc. U. K.* **2020**, *100*, 681–689, doi:10.1017/S0025315420000697.
145. Lakkis, S.; Zeidane, R. Jellyfish Swarm along the Lebanese Coast.; Lebanese Association for the Advancement of Science: University of Beirut, 1991.
146. Li, Y.; Zhou, Z.; Tian, M.; Tian, Y.; Dong, Y.; Li, S.; Liu, W.; He, C. Exploring Single Nucleotide Polymorphism (SNP), Microsatellite (SSR) and Differentially Expressed Genes in the Jellyfish (Rhopilema Esculentum) by Transcriptome Sequencing. *Mar. Genomics* **2017**, *34*, 31–37, doi:10.1016/j.margen.2017.01.007.
147. Lotan, A.; Ben-Hillel, R.; Loya, Y. Life Cycle of Rhopilema Nomadica: A New Immigrant Scyphomedusan in the Mediterranean. *Mar. Biol.* **1992**, *112*, 237–242, doi:10.1007/BF00702467.
148. Lotan, A.; Fine, M.; Benhillel, R. Synchronization of the Life Cycle and Dispersal Pattern of the Tropical Invader Scyphomedusan Rhopilema Nomadica Is Temperature Dependent. *Mar. Ecol. Prog. Ser.* **1994**, *109*, 59–65, doi:10.3354/meps109059.

149. Nishida, S.; Nishikawa, J. *Biodiversity of Marine Zooplankton in Southeast Asia (Project-3: Plankton Group).*; Coastal Marine Science in Southeast Asia–Synthesis Report of the Core University Program of the Japan Society for the Promotion of Science, 2001–2010; Coastal Marine Science Terrapub, Tokyo: Tokyo, Japan, 2011; pp. 59–71;.
150. Ohtsuka, S.; Kondo, Y.; Sakai, Y.; Shimazu, T.; Shimomura, M.; Komai, T.; Yanagi, K.; Fujita, T.; Nishikawa, J.; Miyake, H.; et al. In-Situ Observations of Symbionts on Medusae Occurring in Japan, Thailand, Indonesia and Malaysia. **2010**.
151. Omori, M.; Kitamura, M. Taxonomic Review of Three Japanese Species of Edible Jellyfish (Scyphozoa: Rhizostomeae). *Plankton Biol. Ecol.* **2004**, *51*, 36–51.
152. Ozturk, B.; Isinibilir, M. An Alien Jellyfish Rhopilema Nomadica and Its Impacts to the Eastern Mediterranean Part of Turkey. *J. Black SeaMediterranean Environ.* **2010**, *16*, 149–156.
153. Rumpet, R. *Some Aspects of the Biology and Fishery of Jellyfish Found Along the Coast of Sarawak, Malaysia.*; Department of Fisheries, Ministry of Agriculture: Kuala Lumpur, 1991;
154. Siokou-Frangou, I.; Sarantakos, K.; Epaminondas, D.C. First Record of the Scyphomedusa Rhopilema Nomadica Galil 1990 (Cnidaria: Scyphozoa: Rhizostomeae) in Greece. *Aquat. Invasions* **2006**, *1*, 194–195, doi:http://dx.doi.org/10.3391/ai.2006.1.3.17.
155. Spanier, E.; Galil, B.S. Lessepsian Migration: A Continuous Biogeographical Process. *Endeavour* **1991**, *15*, 102–106, doi:10.1016/0160-9327(91)90152-2.
156. Uchida, T. Distribution of Scyphomedusae in Japanese and Its Adjacent Waters. *J. Fac. Sci. Hokkaido Univ. Ser. VI* **1954**, *12*, 209–219.
157. Ullah, M.S.; Min, G.-S.; Dong, J.; Yoon, W.D.; Choi, J.K. First Record of Rhopilema Esculentum (Scyphozoa, Rhizostomae), Edible Jellyfish in Korea. *Ocean Polar Res.* **2015**, *37*, 287–293, doi:10.4217/OPR.2015.37.4.287.
158. Yusoff, F.M.; Nishikawa, J.; Kuppan, P. Commercial Jellyfish, a Little-Known Fishery Industry in Malaysia. *FishMail* **2010**, *18*, 8–12.
159. Zhu, S.; Ye, M.; Xu, J.; Guo, C.; Zheng, H.; Hu, J.; Chen, J.; Wang, Y.; Xu, S.; Yan, X. Lipid Profile in Different Parts of Edible Jellyfish *Rhopilema Esculentum*. *J. Agric. Food Chem.* **2015**, *63*, 8283–8291, doi:10.1021/acs.jafc.5b03145.
160. Álvarez-Tello, F.J.; López-Martínez, J.; Lluch-Cota, D.B. Trophic Spectrum and Feeding Pattern of Cannonball Jellyfish *Stomolophus Meleagris* (Agassiz, 1862) from Central Gulf of California. *J. Mar. Biol. Assoc. U. K.* **2016**, *96*, 1217–1227, doi:10.1017/S0025315415001605.
161. Calder, D.R. Nematocysts of Stages in the Life Cycle of Stomolophus Meleagris, with Keys to Scyphistomae and Ephyrae of Some Western Atlantic Scyphozoa. *Can. J. Zool.* **1983**, *61*, 1185–1192, doi:https://doi.org/10.1139/z83-161.
162. Camacho-Pacheco, A.V.; Gómez-Salinas, L.C.; Cisneros-Mata, M.Á.; Rodríguez-Félix, D.; Díaz-Tenorio, L.M.; Unzueta-Bustamante, M.L. Feeding

Behavior, Shrinking, and the Role of Mucus in the Cannonball Jellyfish Stomolophus Sp. 2 in Captivity. *Diversity* **2022**, *14*, 103, doi:10.3390/d14020103.
163. Girón-Nava, A.; López-Sagástegui, C.; Aburto-Oropeza, O. On the Conditions of the 2012 Cannonball Jellyfish (Stomolophus Meleagris) Bloom in Golfo de Santa Clara: A Fishery Opportunity? *Fish Manag Ecol* **2015**, *22*, 261–264, doi:https://doi.org/10.1111/fme.1211.
164. Gómez-Salinas, L.C.; López-Martínez, J.; Morandini, A.C. The Young Stages of the Cannonball Jellyfish (Stomolophus Sp. 2) from the Central Gulf of California (Mexico). *Diversity* **2021**, *13*, 229, doi:10.3390/d13060229.
165. Hsieh, Y.-H.P.; Leong, F.; Barnes, K.W. Inorganic Constituents in Fresh and Processed Cannonball Jellyfish (*Stomolophus Meleagris*). *J. Agric. Food Chem.* **1996**, *44*, 3117–3119, doi:10.1021/jf950223m.
166. Huang, Y.A.-W. Cannonball Jellyfish (Stomolophus Meleagris) as a Food Resource. *J. Food Sci.* **1988**, *53*, 341–343, doi:10.1111/j.1365-2621.1988.tb07701.x.
167. Larson, R.J. Diet, Prey Selection and Daily Ration of Stomolophus Meleagris, a Filter-Feeding Scyphomedusa from the NE Gulf of Mexico. *Estuar. Coast. Shelf Sci.* **1991**, *32*, 511–525, doi:10.1016/0272-7714(91)90038-D.
168. López-Martínez, J.; Arzola-Sotelo, E.A.; Nevárez-Martínez, M.O.; Álvarez-Tello, F.J.; Morales-Bojórquez, E. Modeling Growth on the Cannonball Jellyfish Stomolophus Meleagris Based on a Multi-Model Inference Approach. *Hydrobiologia* **2020**, *847*, 1399–1422, doi:10.1007/s10750-020-04182-5.
169. Nevárez-López, C.; Hernández-Saavedra, N.; Sánchez-Paz, A.; Rojas-Posadas, D.; Muhlia-Almazán, A.; López-Martínez, J. Colour Polymorphism and Genetic Structure in the Cannonball Jellyfish (*Stomolophus Meleagris* , L. Agassiz, 1860) in the Gulf of California. *Mar. Biol. Res.* **2020**, *16*, 714–728, doi:10.1080/17451000.2021.1887495.
170. Nevárez-López, C.A.; Sanchez-Paz, A.; Lopez-Martinez, J.; Llera-Herrera, R.; Muhlia-Almazan, A. Metabolic Response of the Cannonball Jellyfish Stomolophus Meleagris upon Short-Term Exposure to Thermal Stress. *J. Sea Res.* **2020**, *166*, 101959, doi:10.1016/j.seares.2020.101959.
171. Padilla-Serrato, J.G.; López-Martínez, J.; Acevedo-Cervantes, A.; Alcántara-Razo, E.; Rábago-Quiroz, C.H. Feeding of the Scyphomedusa Stomolophus Meleagris in the Coastal Lagoon Las Guásimas, Northwest Mexico. **2013**, *23*.
172. Phillips, P.J.; Burke, W.D.; Keener, E.J. Observations on the Trophic Significance of Jellyfishes in Mississippi Sound with Quantitative Data on the Associative Behavior of Small Fishes with Medusae. *Trans. Am. Fish. Soc.* **1969**, *98*, 703–712, doi:10.1577/1548-8659(1969)98[703:OOTTSO]2.0.CO;2.
173. Pico-Vargas, A.; Quirós-Rodríguez, J.; Cedeño-Posso, C. Primer registro de medusas Stomolophus meleagris (Cnidaria: Scyphozoa) en la bahía de Cispatá, Córdoba, Colombia. *Rev. Biol. Mar. Oceanogr.* **2016**, *51*, 709–712, doi:10.4067/S0718-19572016000300024.
174. Reza, M.; Ocampo, L.; Campos-Dávila, L. Association of Three Carangidae Juvenile Fishes with Cannonball Jellyfish *Stomolophus Meleagris* in Bahía de La

Paz, Gulf of California. *Rev. Biol. Mar. Oceanogr.* **2019**, *53*, 387, doi:10.22370/rbmo.2018.53.3.1362.

175. Rountree, R. The Ecology of Stomolophus Meleagris, the Cannon Ball Jellyfish, and Its Symbionts, with Special Emphasis on Behavior. B.S. Marine Biology (honours), University of North Carolina: Wilmington, North Carolina, 1983.
176. Carvalho-Saucedo, L.; García-Domínguez, F.; Rodríguez-Jaramillo, C.; López-Martínez, J. Variación lipídica en los ovocitos de la medusa Stomolophus meleagris (Scyphozoa: Rhizostomeae), durante el desarrollo gonádico, en la laguna Las Guásimas, Sonora, México. *Rev. Biol. Trop.* **2009**, *58*, doi:10.15517/rbt.v58i1.5198.
177. Tahera, Q.; Kazmi, Q.B. New Records of Two Jellyfish Medusae (Cnidaria: Scyphozoa: Catostylidae: Cubozoa: Chirodropidae) from Pakistani Waters. *Mar. Biodivers. Rec.* **2008**, *1*, e30, doi:10.1017/S1755267206002983.
178. Toom, P.M.; Chan, D.S. Preliminary Studies of Nematocysts from the Jellyfish Stomolophus Meleagris. *Toxicon* **1972**, *10*, 605–610, doi:https://doi.org/10.1016/0041-0101(72)90122-5.
179. Tunberg, B.G.; Reed, S.A. Mass Occurrence of the Jellyfish Stomolophus Meleagris and an Associated Spider Crab Libinia Dubia, Eastern Florida. *Fla. Sci.* **2004**, *67*, 93–104.
180. Zhuang, Y.L.; Sun, L.P. Anti-Melanogenic Activities of Collagen Peptides from Jellyfish (Stomolophus Meleagris). *Adv. Mater. Res.* **2011**, *343–344*, 505–512, doi:10.4028/www.scientific.net/AMR.343-344.505.
